# Supplementary material for: gSV: a general structural variant detector using the third-generation sequencing data
Source: Brief Bioinform. 2026 Jun 11;27(3):bbag294. doi: 10.1093/bib/bbag294 (PMC13256234; doi:10.1093/bib/bbag294)
Supplement: gSV_SUPP_bbag294 [file gsv_supp_bbag294.pdf]

Supplementary Materials:
gSV: a general structural variant detector using the third-generation sequencing data

Jingyu Hao[a,1], Jiandong Shi[b,1], Sheng Lian[a], Zhen Zhang[a], Yongyi Luo[b], Taobo Hu[c], Toyotaka Ishibashi[d], Depeng Wang[e], Shu Wang[c,*], Xiaodan Fan[b,*] and Weichuan Yu[a,*]

[a]Department of Electronic and Computer Engineering, Hong Kong University of Science and Technology, Hong Kong SAR, China
[b]Department of Statistics and Data Science, The Chinese University of Hong Kong, Hong Kong SAR, China
[c]Department of Breast Surgery, Peking University People's Hospital, Beijing, China
[d]Division of Life Science, Hong Kong University of Science and Technology, Hong Kong SAR, China
[e]GrandOmics Inc, Beijing, China

1. Supplementary Notes

1.1. Data simulation

Simple SV simulation

To simulate simple structural variants (SVs) more realistically, we utilized the simulator VarSim[1], which can sample from an existing database, to insert four types of simple SV (deletion (DEL), duplication (DUP), insertion (INS), and invertion (INV)) into the reference genome (GRCh37). These SVs were sourced from the Database of Genomic Variants (DGV), which summarizes the SVs in the human genome. We construct the SV set according to the proportion of different SVs in DGV. Since VarSim does not support translocation (TRA), and INVs appear relatively rarely in the DGV database, we increased the proportion of INVs to 4% to better evaluate the detection capabilities of different callers for INVs.

Specifically, we generate 2,700 INSs, 5,700 DELs, 1,530 DUPs, and 400 INVs. However, practical issues such as sampling multiple SVs at the same position limited the generation of some SVs, resulting in the following numbers of simple SVs generated: 2,675 INS, 4,972 DELs, 1,409 DUPs, and 362 INVs. The commands to generate the simulation data are shown as follows:

- `wget http://web.stanford.edu/group/wonglab/varsim/insert_seq.txt`
- `wget http://web.stanford.edu/group/wonglab/varsim/GRCh37_hg19_supportingvariants_2013-07-23.txt`
- `varsim.py --vc_in_vcf vc_file --sv_insert_seq insert_seq.txt --sv_dgv GRCh37_hg19_supportingvariants_2013-07-23.txt --reference hs37d5.fa --id sim --seed 17 --disable_sim --vc_num_snp 0 --vc_num_ins 0 --vc_num_del 0 --vc_num_mnp 0 --vc_num_complex 0 --sv_num_ins 2700 --sv_num_del 5700 --sv_num_dup 1530 --sv_num_inv 400 --sv_percent_novel 0 --sv_min_length_lim 50 --sv_max_length_lim 10000 --nlanes 1 --total_coverage 1 --java_max_mem 50g --simulator_executable $OPT_DIR/ART/art_bin_VanillaIceCream/art_illumina --out_dir out --log_dir log --work_dir work`

Complex SV simulation

We utilized VISOR [2] to construct complex SVs. Firstly, we used the built-in script of VISOR to randomly generate some basic SVs. Taking ID 5 (DUP+INV+DEL) as an example, we first randomly generated duplications.

- `Rscript randomregion.r -d chrom.dim.tsv -n num -l length_mean -s length_sd -x exclude_regions.bed -v 'tandemduplication' -r '100' | sortBed > DUP.bed`

Next, based on the position of each DUP, we manually generated the Browser Extensible Data (BED file) containing the positions of INVs and DELs ('INV_DEL.bed'). Since VISOR does not support repeating insertions of SVs at

*Corresponding author(s).
[1]These authors contributed equally to this work.

overlapping positions, we first utilized ‘DUP.bed’ to insert DUPs into the reference sequence, resulting in Sequence 1. Then, we insert INVs and DUPs into Sequence 1 based on ‘INV_DEL.bed’ and obtained the final sequence.

- `VISOR HACK -g ref.fa -bed DUP.bed -o /path/to/output`
- `VISOR HACK -g s1.fa -bed INV_DEL.bed -o /path/to/output`

It is notable that for the first three combinations (ID 1-3) of complex SVs (no overlapping relationship between sub-SVs), the sequence containing all SVs can be generated at once.

Reads generation

After obtaining the sequences with inserted SVs, we utilized PBSIM2 [3] to simulate Continuous Long Reads (CLRs). The quality scores of reads are generated by the factorized information criteria(FIC)-HMM of P6C4 chemistry.

- `pbsim  -hmm_model data/P6C4.model   sequence.fa`

1.2. Software Version and Parameters

Truvari (V4.1.0) [4]

- `bgzip caller.vcf && tabix caller.vcf.gz`
- `truvari bench -b ground_truth.vcf.gz -c caller.vcf.gz -o dir --passonly -p 0.00 -r 1000 -P 0.5`

Sniffles (V2.2) [5]

- `sniffles -i sample.sorted.bam -v sniffles.vcf -reference reference.fa`

CuteSV (V2.0.3) [6]

- `cuteSV sample.sorted.bam reference.fa cutesv.vcf workdir`

PBSV (V2.9.0) (https://github.com/PacificBiosciences/pbsv/)

- `pbsv discover sample.sorted.bam sample.svsig.gz && pbsv call reference.fa sample.svsig.gzpbsv.vcf`

Debreak (V1.0.2) [7]

- `debreak -bam sample.sorted.bam -outpath debreak -rescue_large_ins -rescue_dup -poa -ref reference.fa`

SVision-Pro (V1.8) [8]

- `SVision-pro --target_path sample.sorted.bam --genome_path reference.fa --model_path {SVISIONPRO_DIR}/src/pre_process/model_liteunet_256_8_16_32_32_32.pth --out_path workdir --sample_name svision-pro --detect_mode germline`

Jasmine (V1.1.5) [9]

- `jasmine file_list=sample_list.txt out_file=Jasmine.merge.vcf`

SURVIVOR (V1.0.7) [10]

- `SURVIVOR sample_list.txt 1000 1 1 1 0 50 SURVIVOR.merge.vcf`

Table 1
Common FLAG meanings in alignment results (SAM file).

Value	Meaning
0	Single-end sequencing, successfully mapped, and aligned to the forward strand.
16	Reverse-complemented relative to the reference genome (aligned to the reverse strand).
2048	Supplementary alignment: part of a chimeric alignment (e.g., split reads spanning distant genomic regions).
2064	Both a supplementary alignment and reverse-complemented.

1.3. Encoding Step in gSV

The encoding step aims to identify all differences between the reference genome and aligned reads, encompassing both sequencing errors and potential variant signatures.

Unlike existing alignment-based SV detection tools, we do not initially specify SV types and build corresponding models. Instead, we identify all the differences between the reference genome and the reads from the beginning. The reference sequence for each chromosome is encoded as a $4 \times reference\_length$ matrix, and the alignment results of each read are encoded as a $4 \times read\_length$ matrix. The start and end positions of each result are also recorded to determine their positions in the reference. By subtracting the two matrices, we obtain all the absolute differences between the reads and the reference. Unlike other approaches that capture signals conforming to predefined models via CIGAR strings, this matrix-based encoding preserves all potential variant information, making it possible to detect novel SVs whose patterns have not yet been summarized in existing models. Moreover, since most bases between the reads and the reference are identical, the resulting matrices are inherently sparse. This sparsity substantially reduces memory usage and computational cost, while enabling efficient parallel processing.

Specifically, the read matrices are constructed by extracting key fields from alignment results stored in the Sequence Alignment/Map (SAM) format, including the FLAG (a bitwise integer encoding alignment properties such as paired-end status and strand orientation), POS (the 1-based leftmost genomic position of the alignment), CIGAR string (a Compact Idiosyncratic Gapped Alignment Report summarizing alignment operations), and SEQ (the nucleotide sequence of the read). The CIGAR string comprises integer-operation pairs where the integer specifies the number of bases, and the operation defines the alignment type: "M" (generic alignment match, encompassing "=" for exact matches and "X" for mismatches), "I" (insertion relative to the reference), "D" (deletion relative to the reference), "S" (soft-clipped unaligned bases), and "H" (hard-clipped unaligned bases). Common FLAG values and their interpretations are detailed in Table 1.

Each sequencing read is encoded into a matrix with four rows corresponding to nucleotides A, T, C, and G. For the simplest case (FLAG = 0), the sequence is extracted from the SEQ field and encoded according to its CIGAR string. For CIGAR operations "M" (including "=" and "X"), each base is represented as a one-hot vector (e.g., $A \rightarrow [1\ 0\ 0\ 0]^T$). For CIGAR "I", the inserted bases are compressed into one column and added to the vector at the insertion position. For example, an 8-base insertion ("8I") $ATCGATCG$ at a C nucleotide position would generate a vector $[2\ 2\ 3\ 2]^T$, calculated as $[2\ 2\ 2\ 2]^T$ (insertion) $+[0\ 0\ 1\ 0]^T$ (insertion position base C). For CIGAR operation "D", the corresponding column is encoded as a zero vector $[0\ 0\ 0\ 0]^T$. Bases corresponding to "S" or "H" are skipped because they are not part of the alignment. For FLAG = 16 (reverse-complement alignment), the SEQ field shows the reverse complement of the original read, but the same encoding approach is employed without additional transformations. Supplementary alignments (marked by FLAG=2048 or 2064) represent chimeric alignment records for a single read that cannot be fully represented by a single linear alignment. These occur when a read is split into multiple segments, resulting in one primary alignment (the highest-confidence alignment) and one or more supplementary alignments, each mapped to distinct regions of the reference genome. To preserve the chimeric relationship inherent in split-read alignments, we encode both the primary alignment and its supplementary counterparts into a single matrix. When supplementary and primary alignments share the same strand orientation, they are placed in the matrix based on their relative position, and the regions between them are represented by 0. For supplementary alignments on the opposing strand relative to the primary alignment, the corresponding segment in the supplementary SEQ is reverse-complemented before encoding to retain strand discordance as a potential variant signal.

Toy Example Demonstration

This section demonstrates how gSV's encoding operates through four toy reads representing the four alignment scenarios in the main text.

Suppose that we have the reference genome sequence $AAGCTTCTCACCCTG$ and four toy reads as follows:

- read 1: $TTCATCGATCGTCCCTG$ (aligned as given)
- read 2: $CAGGGGAATT$ (aligned in reverse complement orientation)
- read 3: $AAGCCCCTG$ (split-aligned)
- read 4: $AAGCTTCTCCAGGGT$ (chimeric aligned)

Figure 1 shows the alignment results where lowercase bases denote clipped regions. The corresponding Sequence Alignment/Map (SAM) format is shown in Figure 2, which is the standard format to record alignment results. Note that BAM files (Binary Alignment/Map), the compressed binary representation of SAM files, serve as the standard input format for our gSV pipeline.

```
Coor     1  2  3  4  5  6  7                          8  9  10 11 12 13 14 15
ref      A  A  G  C  T  T  C  *  *  *  *  *  *  *  *  T  C  A  C  C  C  T  G

+ read1              T  T  C  A  T  C  G  A  T  C  G  T  C  *  *  C  C  T  G
- read2  A  A  *  *  T  T  C                          *  *  *  C  C  C  T  G
+ read3  A  A  G  C  .  .  .                          .  .  .  C  C  C  T  G
+ read4  A  A  G  C  T  T  C                          T  C  c  a  g  g  g  t
- read4                                                     A  C  C  C  T  G  g  a  g  a  a  g  c  t  t
```

Figure 1: Alignment results of four reads. '+' denotes forward strand alignment; '-' denotes reverse complementary strand alignment.

QNAME	FLAG	RNAME	POS	CIGAR	SEQ
read1	0	ref	5	3M8I2M2D4M	TTCATCGATCGTCCCTG
read2	16	ref	1	2M2D3M3D5M	AATTCCCCTG
read3	0	ref	1	4M5S	AAGCCCCTG
read3	2048	ref	11	4S5M	AAGCCCCTG
read4	0	ref	1	9M6S	AAGCTTCTCCAGGGT
read4	2064	ref	10	6M9S	ACCCTGGAGAAGCTT

Figure 2: The alignment results of four reads in SAM format. The QNAME (query name) serves as the identifier for each read; the FLAG indicates alignment information (0: a primary alignment in the forward strand, 16: a reverse complement alignment, 2048: a supplementary alignment on the forward strand, and 2064: a supplementary alignment on the reverse strand); the RNAME (reference name) specifies the reference sequence to which the read is aligned; the POS indicates the starting position of the alignment on the reference, the CIGAR string describes the alignment specifics (M: matches or mismatch, I: insertions, D: deletions, and S :soft clips); and the SEQ field contains the sequence of bases in the read.

Read 1 shows the simplest case. The read is a forward-strand alignment, so the FLAG is 0. It aligns starting at position 5 (POS=5), with an 8-base insertion after position 7, and a 2-base deletion at positions 10-11. So, the CIGAR string is "3M8I2M2D4M", representing 3 matches, 8 insertions, 2 matches, 2 deletions, and 4 matches. We extract the sequence from the SEQ field and encode it according to the CIGAR string, as shown in Figure 3. By comparing this encoded read matrix with the reference matrix, we identify all inconsistencies, that is, potential variant locations (positions 7 and 10-11, highlighted in yellow in Figure 3).

For read 2, we observe that the reverse complement of the read matches the reference (FLAG=16), and the SEQ field of the SAM file already converts the sequence to its reverse complement. In this case, we directly encode the SEQ

Reference: AAGCTTCTCACCCTG

read1: TTCATCGATCGTCCCTG

FLAG:0 POS:5 CIGAR: 3M8I2M2D4M SEQ: TTCATCGATCGTCCCTG

POS	1	2	3	4	5	6	7	8	9	10	11	12	13	14	15
REF	A	A	G	C	T	T	C	T	C	A	C	C	C	T	G
A	1	1	0	0	0	0	0	0	0	1	0	0	0	0	0
T	0	0	0	0	1	1	0	1	0	0	0	0	0	1	0
C	0	0	0	1	0	0	1	0	1	0	1	1	1	0	0
G	0	0	1	0	0	0	0	0	0	0	0	0	0	0	1
READ					T	T	C*	T	C			C	C	T	G
A					0	0	2	0	0	0	0	0	0	0	0
T					1	1	2	1	0	0	0	0	0	1	0
C					0	0	3	0	1	0	0	1	1	0	0
G					0	0	2	0	0	0	0	0	0	0	1
Diff					T	T	C	T	C	A	C	C	C	T	G
A					0	0	2	0	0	1	0	0	0	0	0
T					0	0	2	0	0	0	0	0	0	0	0
C					0	0	2	0	0	0	1	0	0	0	0
G					0	0	2	0	0	0	0	0	0	0	0

Figure 3: Diagram of encoding method for read with FLAG=0. The red box shows the encoding process of CIGAR "I", the inserted bases are compressed into one column and added to the vector at the insertion position. In this example, the 8-base insertion ("8I") at a C nucleotide position would generate a vector $[2\ 2\ 3\ 2]^T$, calculated as $[2\ 2\ 2\ 2]^T$ (insertion) $+[0\ 0\ 1\ 0]^T$ (insertion position base C). The blue box shows "D", the corresponding column is encoded as a zero vector. "Diff" represents the absolute difference between the REF matrix and the READ matrix. After subtraction, all the differences (highlighted in yellow) will be displayed.

string into the matrix, as shown in Figure 4. The encoding method for "I" and "D" in the CIGAR string is the same as when FLAG=0 (in this example, the CIGAR string only contains "D").

For reads 3 and 4, the reads are split into segments recorded as separate SAM lines. One of them is marked as primary alignment, and the additional alignments of the read are marked as supplementary alignments. To preserve the chimeric relationship inherent in split-read alignments, we encode both the primary alignment and its supplementary counterparts into a single matrix.

For read 3, the supplementary and primary alignments share the same strand orientation. Thus, the FLAG for the primary alignment is 0 (forward), and the FLAG for the supplementary alignment is 2048 (forward), as shown in Figure 2, read 3. Alternatively, the primary alignment is 16 (reverse complementary), and the supplementary alignment is 2064 (reverse complementary). Then, we position them according to relative genomic coordinates, representing the intervening regions with zeros. The encoding results of read 3 is shown in Figure 5.

For read 4, the supplementary alignments are on the opposite strand relative to the primary alignment (the FLAG for the primary alignment is 0 and for the supplementary alignment is 2064, or the FLAG for the primary alignment is 16 and for the supplementary alignment is 2048), indicating an INV. From Figure 2, read 4, we can see that the SEQ records for these two segments are different and complement each other. If we encode them directly, the matrix will be the same as the reference matrix, as shown at the bottom of Figure 6, yielding a null difference matrix and losing the INV information. Therefore, we use the primary direction as the benchmark and convert the SEQ string of supplementary alignments into their reverse complements. In other words, we use the original sequence of read 4 to encode the supplementary part instead of using the string recorded in the SEQ field. The middle part of Figure 6 shows our method. This preserved the INVs (highlighted in yellow) after matrix subtraction.

Reference: AAGCTTCTCACCCTG
read2: CAGGGGAATT
FLAG:16 POS:1 CIGAR: 2M2D3M3D5M SEQ: AATTCCCCTG

POS	1	2	3	4	5	6	7	8	9	10	11	12	13	14	15
REF	A	A	G	C	T	T	C	T	C	A	C	C	C	T	G
A	1	1	0	0	0	0	0	0	0	1	0	0	0	0	0
T	0	0	0	0	1	1	0	1	0	0	0	0	0	1	0
C	0	0	0	1	0	0	1	0	1	0	1	1	1	0	0
G	0	0	1	0	0	0	0	0	0	0	0	0	0	0	1
READ	A	A			T	T	C				C	C	C	T	G
A	1	1	0	0	0	0	0	0	0	0	0	0	0	0	0
T	0	0	0	0	1	1	0	0	0	0	0	0	0	1	0
C	0	0	0	0	0	0	1	0	0	0	1	1	1	0	0
G	0	0	0	0	0	0	0	0	0	0	0	0	0	0	1
Diff	A	A	G	C	T	T	C	T	C	A	C	C	C	T	G
A	0	0	0	0	0	0	0	0	0	1	0	0	0	0	0
T	0	0	0	0	0	0	0	1	0	0	0	0	0	0	0
C	0	0	0	1	0	0	0	0	1	0	1	0	0	0	0
G	0	0	1	0	0	0	0	0	0	0	0	0	0	0	0

Figure 4: Diagram of encoding method for read with FLAG=16. The blue box shows "D", the corresponding column is encoded as a zero vector $[0\ 0\ 0\ 0]^T$. "Diff" represents the absolute difference between the REF matrix and the READ matrix. After subtraction, all the differences (highlighted in yellow) will be displayed.

Reference: AAGCTTCTCACCCTG
read3: AAGCCCCTG
Primary:
FLAG:0 POS:1 CIGAR: 4M5S SEQ: AAGCCCCTG
Supplementary:
FLAG:2048 POS:11 CIGAR: 4S5M SEQ: AAGCCCCTG

POS	1	2	3	4	5	6	7	8	9	10	11	12	13	14	15
REF	A	A	G	C	T	T	C	T	C	A	C	C	C	T	G
A	1	1	0	0	0	0	0	0	0	1	0	0	0	0	0
T	0	0	0	0	1	1	0	1	0	0	0	0	0	1	0
C	0	0	0	1	0	0	1	0	1	0	1	1	1	0	0
G	0	0	1	0	0	0	0	0	0	0	0	0	0	0	1
READ	A	A	G	C							C	C	C	T	G
A	1	1	0	0	0	0	0	0	0	0	0	0	0	0	0
T	0	0	0	0	0	0	0	0	0	0	0	0	0	1	0
C	0	0	0	1	0	0	0	0	0	0	1	1	1	0	0
G	0	0	1	0	0	0	0	0	0	0	0	0	0	0	1
Diff	A	A	G	C	T	T	C	T	C	A	C	C	C	T	G
A	0	0	0	0	0	0	0	0	0	1	0	0	0	0	0
T	0	0	0	0	1	1	0	1	0	0	0	0	0	0	0
C	0	0	0	0	0	0	1	0	1	0	0	0	0	0	0
G	0	0	0	0	0	0	0	0	0	0	0	0	0	0	0

Figure 5: Diagram of encoding method for reads with supplementary alignment on the same strand. The green box highlights the encoding results of primary alignment, and the purple box highlights the results of supplementary alignment. The intervening regions are encoding with zeros. After subtraction, all the differences (highlighted in yellow) will be displayed.

Reference: AAGCTTCTCACCCTG

read4: AAGCTTCTCCAGGGT

Primary:

FLAG:0 POS:1 CIGAR: 9M6S SEQ: AAGCTTCTCCAGGGT

Supplementary:

FLAG:2064 POS:10 CIGAR: 6M9S SEQ: ACCCTGGAGAAGCTT

POS	1	2	3	4	5	6	7	8	9	10	11	12	13	14	15
REF	A	A	G	C	T	T	C	T	C	A	C	C	C	T	G
A	1	1	0	0	0	0	0	0	0	1	0	0	0	0	0
T	0	0	0	0	1	1	0	1	0	0	0	0	0	1	0
C	0	0	0	1	0	0	1	0	1	0	1	1	1	0	0
G	0	0	1	0	0	0	0	0	0	0	0	0	0	0	1

Converting the supplements to their reverse complementary

READ	A	A	G	C	T	T	C	T	C	C	A	G	G	G	T
A	1	1	0	0	0	0	0	0	0	0	1	0	0	0	0
T	0	0	0	0	1	1	0	1	0	0	0	0	0	0	1
C	0	0	0	1	0	0	1	0	1	1	0	0	0	0	0
G	0	0	1	0	0	0	0	0	0	0	0	1	1	1	0
Diff	A	A	G	C	T	T	C	T	C	A	C	C	C	T	G
A	0	0	0	0	0	0	0	0	0	1	1	0	0	0	0
T	0	0	0	0	0	0	0	0	0	0	0	0	0	1	1
C	0	0	0	0	0	0	0	0	0	1	1	1	1	0	0
G	0	0	0	0	0	0	0	0	0	0	0	1	1	1	1

Without converting the supplements to their reverse complementary

Null difference matrix → Losing variants information

READ	A	A	G	C	T	T	C	T	C	A	C	C	C	T	G
A	1	1	0	0	0	0	0	0	0	1	0	0	0	0	0
T	0	0	0	0	1	1	0	1	0	0	0	0	0	1	0
C	0	0	0	1	0	0	1	0	1	0	1	1	1	0	0
G	0	0	1	0	0	0	0	0	0	0	0	0	0	0	1
Diff	A	A	G	C	T	T	C	T	C	A	C	C	C	T	G
A	0	0	0	0	0	0	0	0	0	0	0	0	0	0	0
T	0	0	0	0	0	0	0	0	0	0	0	0	0	0	0
C	0	0	0	0	0	0	0	0	0	0	0	0	0	0	0
G	0	0	0	0	0	0	0	0	0	0	0	0	0	0	0

Figure 6: Diagram of encoding method for reads with supplementary alignment on the different strand. The green box shows the encoding results of primary alignment, and the purple box shows the results of supplementary alignment. Here, we compare the encoding results of employing reverse-complement sequence vs. native-strand sequence for supplementary SEQ when the supplementary alignment resides on the complementary strand relative to the primary alignment. Without converting the supplements to their reverse complement, we can see that the Diff matrix is null and the variant information is missing.

1.4. Output of gSV

The output of gSV consists of two files: gSV_complex.vcf and gSV.vcf. The gSV_complex.vcf file reports only complex SVs as unified events (Figure 7), whereas gSV.vcf reports both simple SVs and the decomposed components of complex SVs to ensure compatibility with downstream analyses (Figure 8).

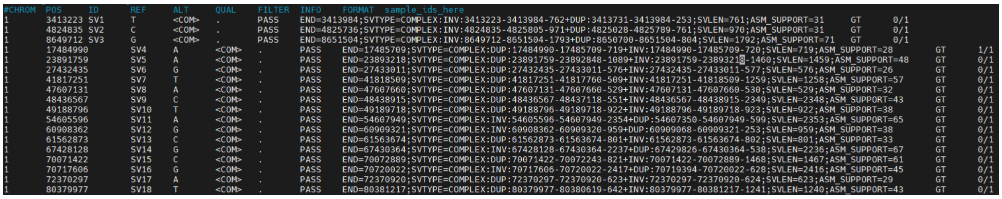

```
#CHROM  POS     ID      REF     ALT     QUAL    FILTER  INFO    FORMAT  sample_ids_here
1       3413223 SV1     T       <COM>   .       PASS    END=3413984;SVTYPE=COMPLEX:INV:3413223-3413984-762+DUP:3413731-3413984-253;SVLEN=761;ASM_SUPPORT=31     GT      0/1
1       4824835 SV2     C       <COM>   .       PASS    END=4825736;SVTYPE=COMPLEX:INV:4824835-4825805-971+DUP:4825028-4825789-761;SVLEN=970;ASM_SUPPORT=31     GT      0/1
1       8649712 SV3     G       <COM>   .       PASS    END=8651504;SVTYPE=COMPLEX:INV:8649712-8651504-1793+DUP:8650700-8651504-804;SVLEN=1792;ASM_SUPPORT=71   GT      0/1
1       17484990        SV4     A       <COM>   .       PASS    END=17485709;SVTYPE=COMPLEX:DUP:17484990-17485709-719+INV:17484990-17485709-720;SVLEN=719;ASM_SUPPORT=28        GT      1/1
1       23891759        SV5     A       <COM>   .       PASS    END=23893218;SVTYPE=COMPLEX:DUP:23891759-23892848-1089+INV:23891759-23893218-1460;SVLEN=1459;ASM_SUPPORT=48     GT      0/1
1       27432435        SV6     G       <COM>   .       PASS    END=27433011;SVTYPE=COMPLEX:DUP:27432435-27433011-576+INV:27432435-27433011-577;SVLEN=576;ASM_SUPPORT=26        GT      0/1
1       41817251        SV7     T       <COM>   .       PASS    END=41818509;SVTYPE=COMPLEX:DUP:41817251-41817760-509+INV:41817251-41818509-1259;SVLEN=1258;ASM_SUPPORT=57      GT      0/1
1       47607131        SV8     A       <COM>   .       PASS    END=47607660;SVTYPE=COMPLEX:DUP:47607131-47607660-529+INV:47607131-47607660-530;SVLEN=529;ASM_SUPPORT=32        GT      0/1
1       48436567        SV9     C       <COM>   .       PASS    END=48438915;SVTYPE=COMPLEX:DUP:48436567-48437118-551+INV:48436567-48438915-2349;SVLEN=2348;ASM_SUPPORT=43      GT      0/1
1       49188796        SV10    T       <COM>   .       PASS    END=49189718;SVTYPE=COMPLEX:DUP:49188796-49189718-922+INV:49188796-49189718-923;SVLEN=922;ASM_SUPPORT=38        GT      0/1
1       54605596        SV11    A       <COM>   .       PASS    END=54607949;SVTYPE=COMPLEX:INV:54605596-54607949-2354+DUP:54607350-54607949-599;SVLEN=2353;ASM_SUPPORT=65      GT      0/1
1       60908362        SV12    G       <COM>   .       PASS    END=60909321;SVTYPE=COMPLEX:INV:60908362-60909320-959+DUP:60909068-60909321-253;SVLEN=959;ASM_SUPPORT=38        GT      0/1
1       61562873        SV13    C       <COM>   .       PASS    END=61563674;SVTYPE=COMPLEX:DUP:61562873-61563674-801+INV:61562873-61563674-802;SVLEN=801;ASM_SUPPORT=33        GT      0/1
1       67428128        SV14    G       <COM>   .       PASS    END=67430364;SVTYPE=COMPLEX:INV:67428128-67430364-2237+DUP:67429826-67430364-538;SVLEN=2236;ASM_SUPPORT=67      GT      0/1
1       70071422        SV15    C       <COM>   .       PASS    END=70072889;SVTYPE=COMPLEX:DUP:70071422-70072243-821+INV:70071422-70072889-1468;SVLEN=1467;ASM_SUPPORT=61      GT      0/1
1       70717606        SV16    G       <COM>   .       PASS    END=70720022;SVTYPE=COMPLEX:INV:70717606-70720022-2417+DUP:70719394-70720022-628;SVLEN=2416;ASM_SUPPORT=45      GT      0/1
1       72370297        SV17    A       <COM>   .       PASS    END=72370920;SVTYPE=COMPLEX:DUP:72370297-72370920-623+INV:72370297-72370920-624;SVLEN=623;ASM_SUPPORT=29        GT      0/1
1       80379977        SV18    T       <COM>   .       PASS    END=80381217;SVTYPE=COMPLEX:DUP:80379977-80380619-642+INV:80379977-80381217-1241;SVLEN=1240;ASM_SUPPORT=43      GT      0/1
```

Figure 7: Screenshot of the gSV_complex.vcf output file showing unified representations of complex SVs.

In gSV_complex.vcf, the details of complex SVs are annotated in the SVTYPE field using the format: COMPLEX: subSV1(type-start-end-length); subSV2(type-start-end-length). For example, SV1 in Figure 7 represents a complex SV composed of an INV spanning positions 3,413,223 to 3,413,984 (length: 762 bp), together with a DUP spanning positions 3,413,731 to 3,413,984 (length: 253 bp). The corresponding decomposed records are reported in gSV.vcf as SV2 and SV3. Notably, gSV.vcf includes not only the components of complex SVs but also all detected simple SVs, such as SV1 in Figure 8.

In addition, different support metrics are reported depending on whether a candidate region enters the complex mode. For candidate regions processed without local assembly, the number of reads supporting the corresponding SV signature is recorded in the INFO field as SUPPORT. In contrast, candidate regions entering the complex mode are processed through local assembly and realignment of reads within the region. Because reads collected from the candidate region may not necessarily span or directly support the final detected SV, it is difficult to accurately define SV-supporting read counts in this scenario. Therefore, for SVs detected from regions processed in complex mode, gSV reports the number of reads used for local assembly in the INFO field as ASM_SUPPORT.

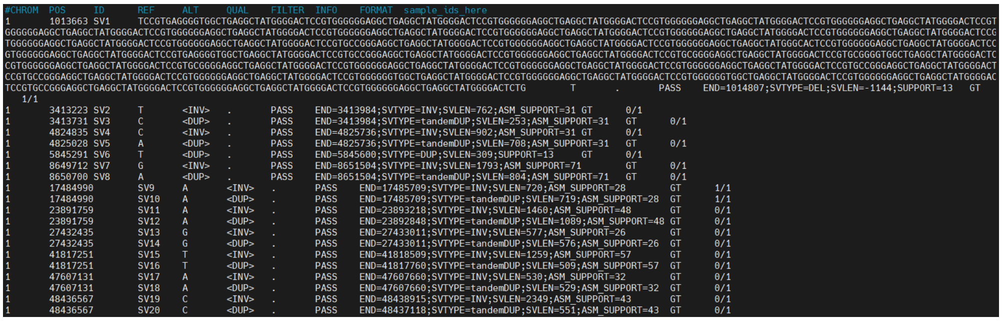

```
#CHROM  POS     ID      REF     ALT     QUAL    FILTER  INFO    FORMAT  sample_ids_here
1       1013663 SV1     TCCGTGAGGGGTGGCTGAGGCTATGGGGACTCCGTGGGGGGAGGCTGAGGCTATGGGGACTCCGTGGGGGGAGGCTGAGGCTATGGGGACTCCGTGGGGGGAGGCTGAGGCTATGGGGACTCCGTGGGGGGAGGCTGAGGCTATGGGGACTCCGT
GGGGGGAGGCTGAGGCTATGGGGACTCCGTGGGGGGAGGCTGAGGCTATGGGGACTCCGTGGGGGGAGGCTGAGGCTATGGGGACTCCGTGGGGGGAGGCTGAGGCTATGGGGACTCCGTGGGGGGAGGCTGAGGCTATGGGGACTCCGTGGGGGGAGGCTGAGGCTATGGGGACTCCG
TGGGGGGAGGCTGAGGCTATGGGGACTCCGTGGGGGGAGGCTGAGGCTATGGGGACTCCGTGCCGGGAGGCTGAGGCTATGGGGACTCCGTGGGGGGAGGCTGAGGCTATGGGGACTCCGTGGGGGGAGGCTGAGGCTATGGGCACTCCGTGGGGGGAGGCTGAGGCTATGGGGACTCC
GTGGGGGGAGGCTGAGGCTATGGGGACTCCGTGAGGGGTGGCTGAGGCTATGGGGACTCCGTGCCGGGAGGCTGAGGCTATGGGGACTCCGTGGGGGGAGGCTGAGGCTATGGGGACTCCGTGCGGGGAGGCTGAGGCTATGGGGACTCCGTGCGGGGTGGCTGAGGCTATGGGGACTC
CGTGGGGGGAGGCTGAGGCTATGGGGACTCCGTGCGGGGAGGCTGAGGCTATGGGGACTCCGTGGGGGGAGGCTGAGGCTATGGGGACTCCGTGGGGGGAGGCTGAGGCTATGGGGACTCCGTGGGGGGAGGCTGAGGCTATGGGGACTCCGTGCCGGGAGGCTGAGGCTATGGGGACT
CCGTGCCGGGAGGCTGAGGCTATGGGGACTCCGTGGGGGGAGGCTGAGGCTATGGGGACTCCGTGGGGGGTGGCTGAGGCTATGGGGACTCCGTGGGGGGAGGCTGAGGCTATGGGGACTCCGTGGGGGGTGGCTGAGGCTATGGGGACTCCGTGGGGGGAGGCTGAGGCTATGGGGAC
TCCGTGCCGGGAGGCTGAGGCTATGGGGACTCCGTGGGGGGAGGCTGAGGCTATGGGGACTCTG        T       .       PASS    END=1014807;SVTYPE=DEL;SVLEN=-1144;SUPPORT=13   GT
   1/1
1       3413223 SV2     T       <INV>   .       PASS    END=3413984;SVTYPE=INV;SVLEN=762;ASM_SUPPORT=31 GT      0/1
1       3413731 SV3     C       <DUP>   .       PASS    END=3413984;SVTYPE=tandemDUP;SVLEN=253;ASM_SUPPORT=31   GT      0/1
1       4824835 SV4     C       <INV>   .       PASS    END=4825736;SVTYPE=INV;SVLEN=902;ASM_SUPPORT=31 GT      0/1
1       4825028 SV5     A       <DUP>   .       PASS    END=4825736;SVTYPE=tandemDUP;SVLEN=708;ASM_SUPPORT=31   GT      0/1
1       5845291 SV6     T       <DUP>   .       PASS    END=5845600;SVTYPE=DUP;SVLEN=309;SUPPORT=13     GT      0/1
1       8649712 SV7     G       <INV>   .       PASS    END=8651504;SVTYPE=INV;SVLEN=1793;ASM_SUPPORT=71        GT      0/1
1       8650700 SV8     A       <DUP>   .       PASS    END=8651504;SVTYPE=tandemDUP;SVLEN=804;ASM_SUPPORT=71   GT      0/1
1       17484990        SV9     A       <INV>   .       PASS    END=17485709;SVTYPE=INV;SVLEN=720;ASM_SUPPORT=28        GT      1/1
1       17484990        SV10    A       <DUP>   .       PASS    END=17485709;SVTYPE=tandemDUP;SVLEN=719;ASM_SUPPORT=28  GT      1/1
1       23891759        SV11    A       <INV>   .       PASS    END=23893218;SVTYPE=INV;SVLEN=1460;ASM_SUPPORT=48       GT      0/1
1       23891759        SV12    A       <DUP>   .       PASS    END=23892848;SVTYPE=tandemDUP;SVLEN=1089;ASM_SUPPORT=48 GT      0/1
1       27432435        SV13    G       <INV>   .       PASS    END=27433011;SVTYPE=INV;SVLEN=577;ASM_SUPPORT=26        GT      0/1
1       27432435        SV14    G       <DUP>   .       PASS    END=27433011;SVTYPE=tandemDUP;SVLEN=576;ASM_SUPPORT=26  GT      0/1
1       41817251        SV15    T       <INV>   .       PASS    END=41818509;SVTYPE=INV;SVLEN=1259;ASM_SUPPORT=57       GT      0/1
1       41817251        SV16    T       <DUP>   .       PASS    END=41817760;SVTYPE=tandemDUP;SVLEN=509;ASM_SUPPORT=57  GT      0/1
1       47607131        SV17    A       <INV>   .       PASS    END=47607660;SVTYPE=INV;SVLEN=530;ASM_SUPPORT=32        GT      0/1
1       47607131        SV18    A       <DUP>   .       PASS    END=47607660;SVTYPE=tandemDUP;SVLEN=529;ASM_SUPPORT=32  GT      0/1
1       48436567        SV19    C       <INV>   .       PASS    END=48438915;SVTYPE=INV;SVLEN=2349;ASM_SUPPORT=43       GT      0/1
1       48436567        SV20    C       <DUP>   .       PASS    END=48437118;SVTYPE=tandemDUP;SVLEN=551;ASM_SUPPORT=43  GT      0/1
```

Figure 8: Screenshot of the gSV.vcf output file showing both simple SVs and decomposed components of complex events.

1.5. Statistical Significance Assessment of F1-score Differences

To assess statistical significance, we applied a bootstrap procedure to estimate the 95% confidence intervals of the F1-score differences for DELs, DUPs, and INVs. Let $\Delta F_1 = F_1^{\mathrm{gSV}} - F_1^{\mathrm{best}}$.

Taking DELs as an example, let n denote the number of ground truth DELs. We sample n variants with replacement from the union of the ground truth set, the gSV call set, and the call set of the best-performing existing method. Based on each resampled dataset, precision and recall are computed for both methods, from which the F1-scores and their difference ΔF_1 are derived. By repeating the above steps 1,000 times, we can obtain the bootstrap distribution of the ΔF_1 and the 95% confidence interval.

The resulting 95% confidence intervals for DELs, DUPs, and INVs are [0.275%, 1.083%], [1.901%, 4.270%], and [1.151%, 6.726%], respectively. None of these intervals include 0, indicating that the improvements of gSV over the best-performing existing method are statistically significant.

2. Supplementary Table

The 28 genes in the Breast cancer dataset are listed in Supplementary Table 1.

Supplementary Table 1

The 28 genes of focus related to breast cancer.

Chrom	Start	End	Gene	Length
chr2	211,375,717	212,538,802	ERBB4	1,163,085
chr2	214,725,645	214,809,711	BARD1	84,066
chr3	179,148,114	179,240,093	PIK3CA	91,979
chr5	132,556,924	132,644,621	RAD50	87,697
chr6	151,654,148	152,129,604	ESR1	475,456
chr7	55,019,017	55,211,628	EGFR	192,611
chr8	31,033,262	31,175,871	WRN	142,609
chr8	89,933,336	89,984,724	NBS1	51,388
chr8	144,511,284	144,517,828	RECQL4	6,544
chr9	95,099,054	95,317,730	FANCC	218,676
chr11	94,415,570	94,512,701	MRE11	97,131
chr11	101,029,624	101,130,681	PGR	101,057
chr11	108,222,484	108,369,099	ATM	146,615
chr12	56,080,025	56,103,507	ERBB3	23,482
chr13	32,315,480	32,399,672	BRCA2	84,192
chr14	67,819,779	68,683,118	RAD51B	863,339
chr14	64,226,712	64,338,631	ESR2	111,919
chr15	90,717,327	90,816,166	BLM	98,839
chr16	23,603,162	23,641,357	PALB2	38,195
chr16	68,737,290	68,835,542	CDH1	98,252
chr17	31,094,927	31,377,677	NF1	282,750
chr17	35,099,792	35,119,869	RAD51D	20,077
chr17	39,688,084	39,728,662	ERBB2	40,578
chr17	43,044,295	43,125,483	BRCA1	81,188
chr17	58,692,140	58,735,611	RAD51C	43,471
chr17	61,679,186	61,864,120	BRIP1	184,934
chr17	75,626,845	75,667,202	RECQL5	40,357
chr22	28,687,743	28,741,866	CHEK2	54,123

Supplementary Table 2

SVs detected in in-house breast cancer data only by gSV.

Chrom	Start	Gene	Length	Type	Other Information
chr2	211689991	ERBB4	170	DUP	
chr2	211944066	ERBB4	148	DUP	
chr2	212191391	ERBB4	214	DUP	
chr7	55167022	EGFR	754	DUP	Other tools detect this DUP as an INS. Panel (c) of Figure 5 in main text shows the detail.
chr7	55176724	EGFR	246	DUP	
chr14	68037106	RAD51B	84	DEL	
chr15	90804992	BLM	935	DEL	
chr17	61787317	BRIP1	57	DUP	Other tools detect this DUP as an INS.

3. Supplementary Figures

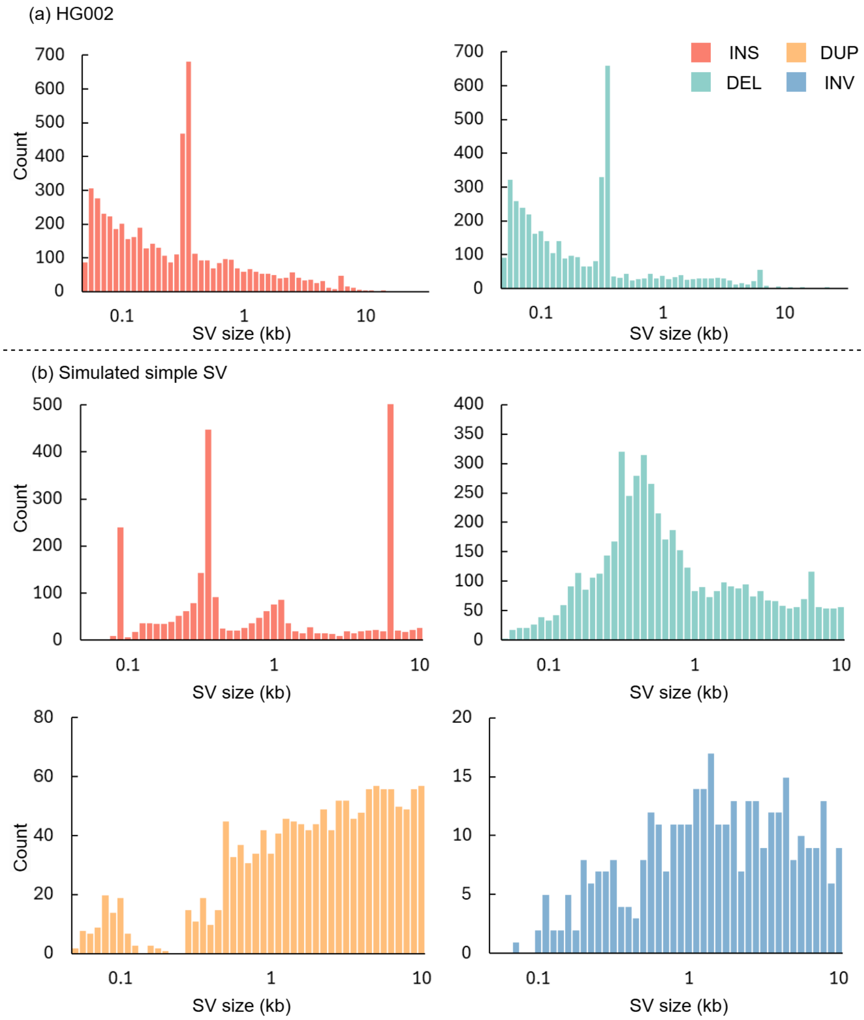

Supplementary Figure 1: The size distributions of simple SVs. The horizontal axis represents the SV length in kilobases, while the vertical axis shows the number of SVs at each length.

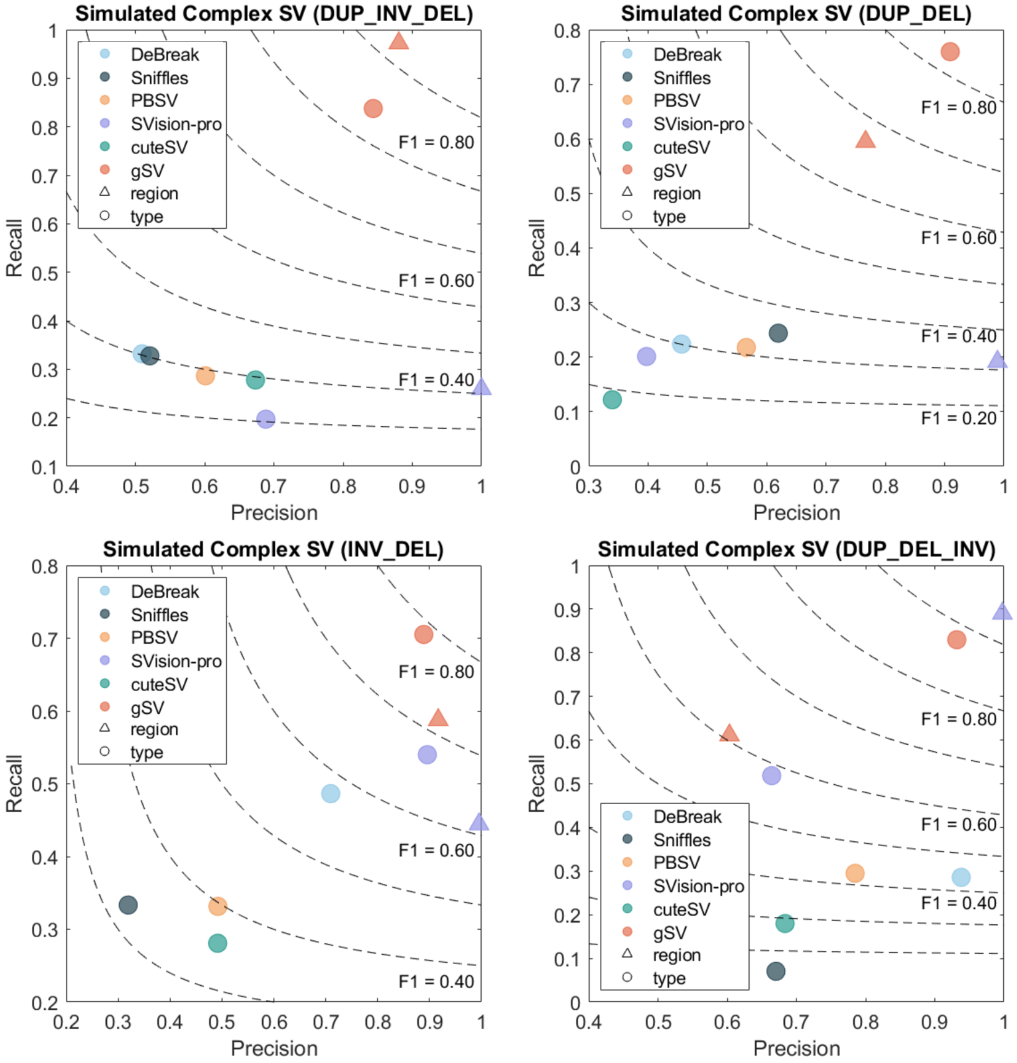

Supplementary Figure 2: The performance of different methods in detecting different types of complex SVs by evaluating the region and type, respectively. Four out of five existing tools (DeBreak, Sniffles, PBSV, and cuteSV) without complex SV detection capability perform worse than gSV in identifying such composite events involving multiple breakpoints. The fifth existing tool SVision-pro performs better than gSV in identifying region loations for DUP_DEL_INV type of SVs. But gSV achieves more accurate type detection than SVision-pro.

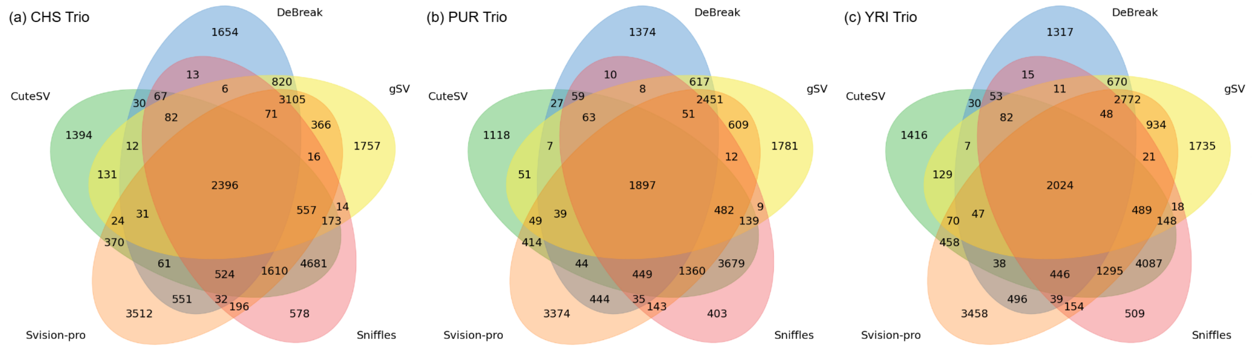

Supplementary Figure 3: Venn diagrams showing the overlap of Mendelian-consistent SVs detected by different tools in two trios: the Southern Han Chinese trio (a) (HG00514/HG00512/HG00513; 26.2x/30.8x/45.3x) and the Puerto Rican trio (b) (HG00733/HG00731/HG00732; 35.5x/35.0x/21.4x).

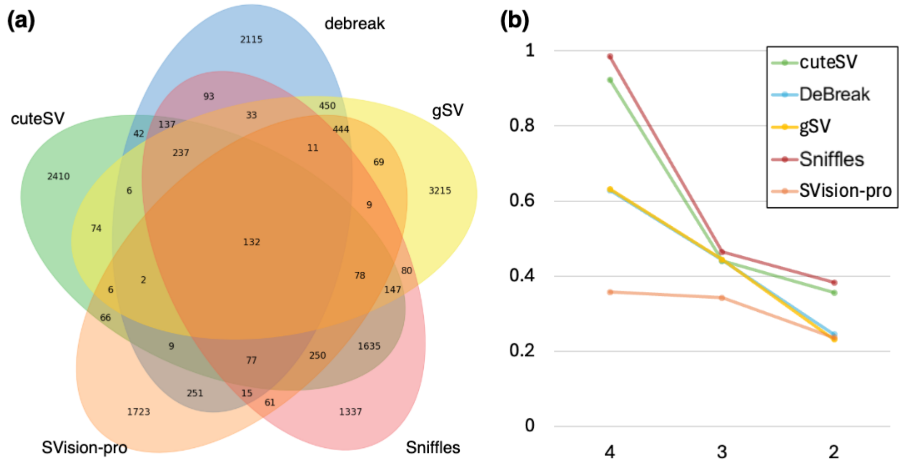

Supplementary Figure 4: Panel (a) shows a Venn diagram of the numbers of somatic SVs detected by different tools on HCC1395. Panel (b) reports the ratios of five tools in SV call sets of different confidence levels. The horizontal axis denotes the number of other tools that have co-detected each SV set. SVs co-detected by four other tools have a higher confidence level than those detected by only three other tools. It can be seen that the curves for all five tools exhibited descending trends as confidence levels decreased, indicating that all tools report more SVs for input sets with higher signal-to-noise ratio (i.e., higher percent of true SVs). Note that the relative height comparison between different lines is meaningless because we do not know the correct ratio of signals within each set, but steeper deceasing means the corresponding tool is more hesitating to report SVs for input set containing less percent of true SVs. Thus gSV, indicated by the constantly sharp decreasing from 4 to 3 and from 3 to 2, may be the most immune tool against noise (i.e., true negatives). From this logic, we argue that the 3215 SVs only reported by gSV may be largely true SVs.

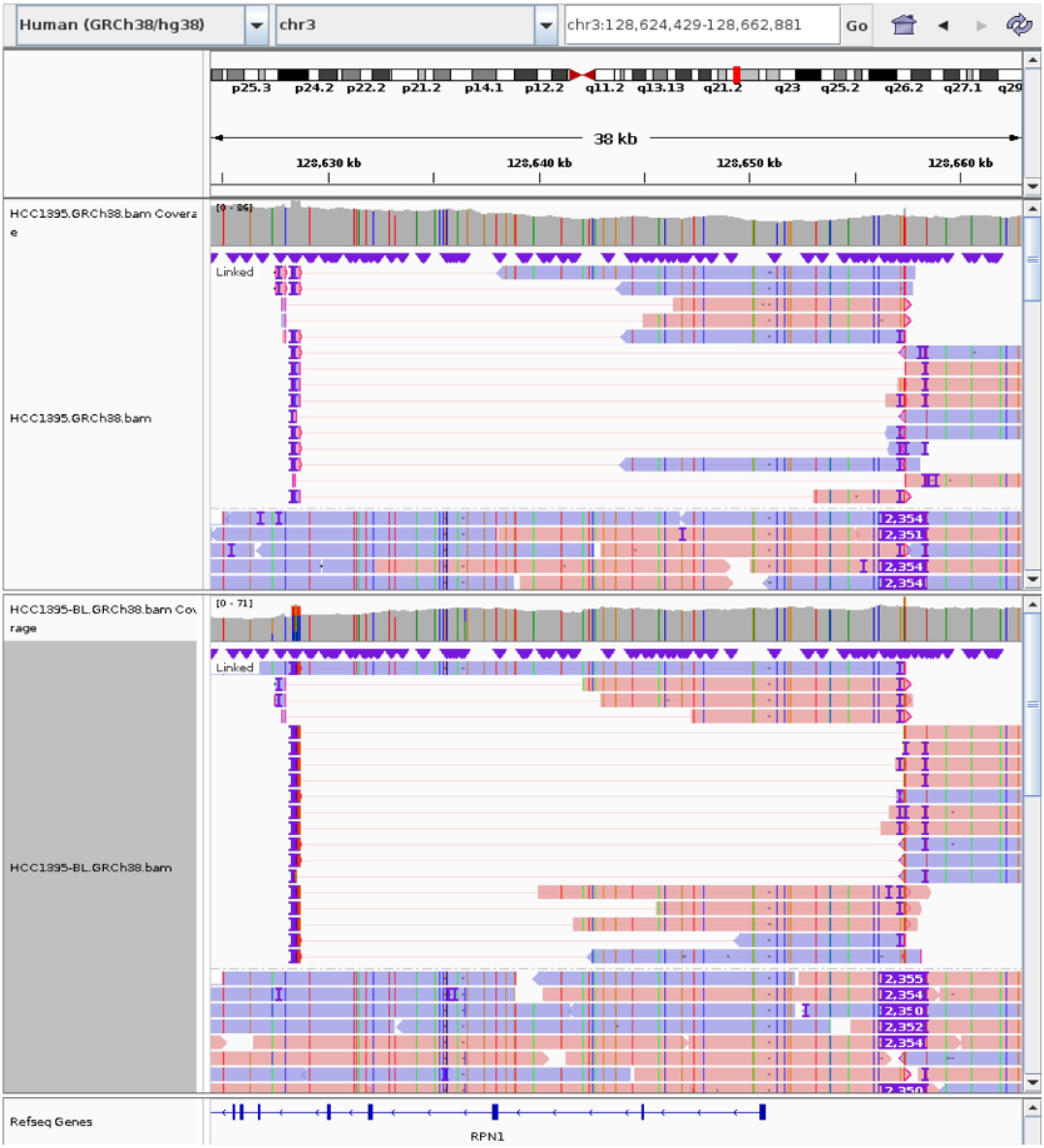

Supplementary Figure 5: A germline INV uniquely detected by gSV in HCC1395 and HCC1954. The INV is located within the RPN1 exonic region. Previous research [11] has demonstrated that RPN1 promotes the proliferation, migration, and invasion of breast cancer cells through the PI3K/AKT/mTOR signaling pathway.

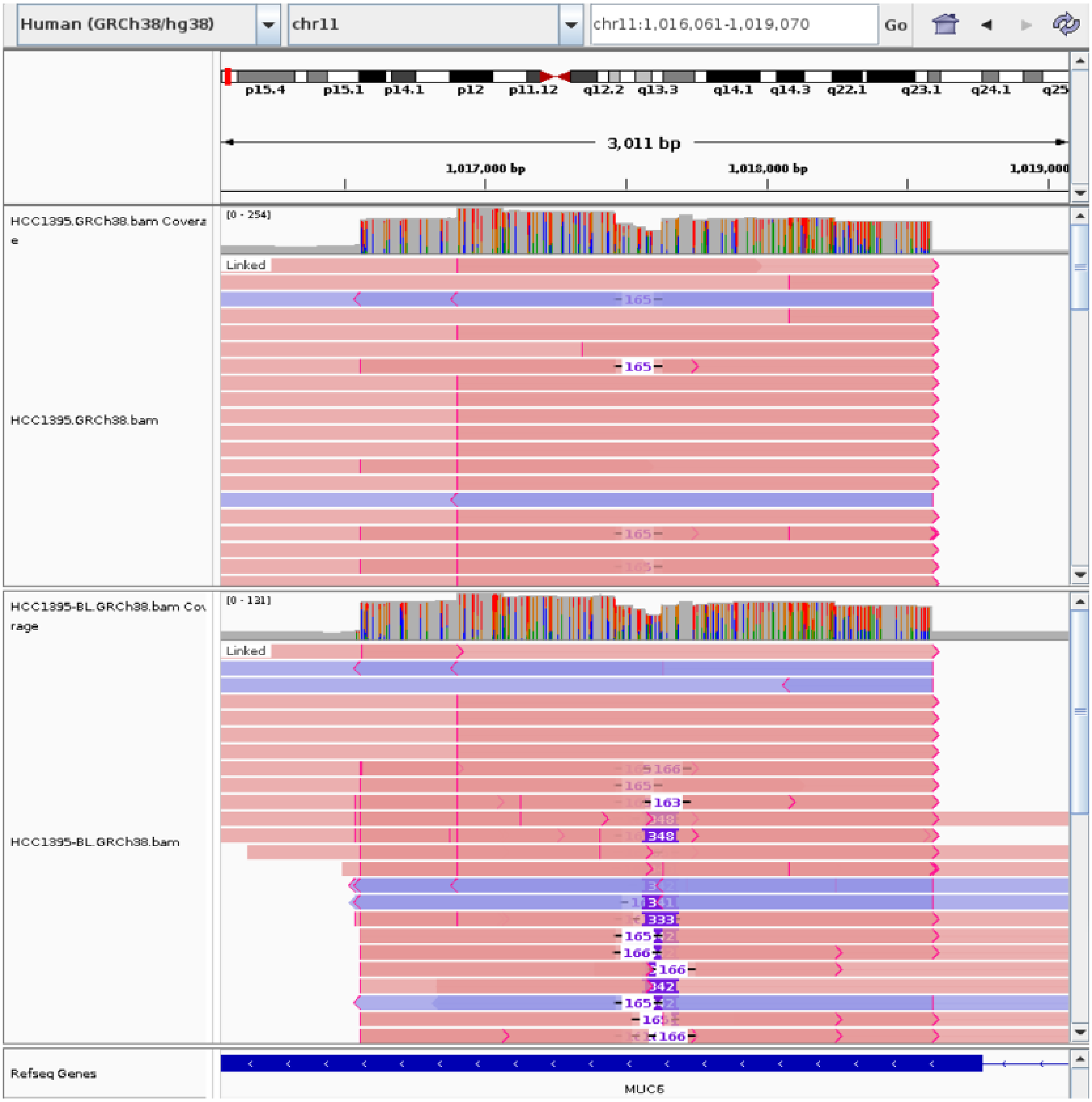

Supplementary Figure 6: Two overlapping DUPs in the exonic region of MUC6 uniquely detected by gSV in HCC1395. Two-step signals in the IGV visualization confirm the findings, whereas competing tools (cuteSV, DeBreak, Sniffles) detected only the second DUP, and SVision-pro failed to identify either. MUC6 is one of five secreted gel-forming mucins (MUC2, MUC5AC, MUC5B, MUC6, and MUC19) expressed from a gene cluster at chromosome 11p15. Literature review reveals that MUC6 alterations have been linked to tumor aggressiveness in cancers [12], and it is overexpressed in breast cancer [13].

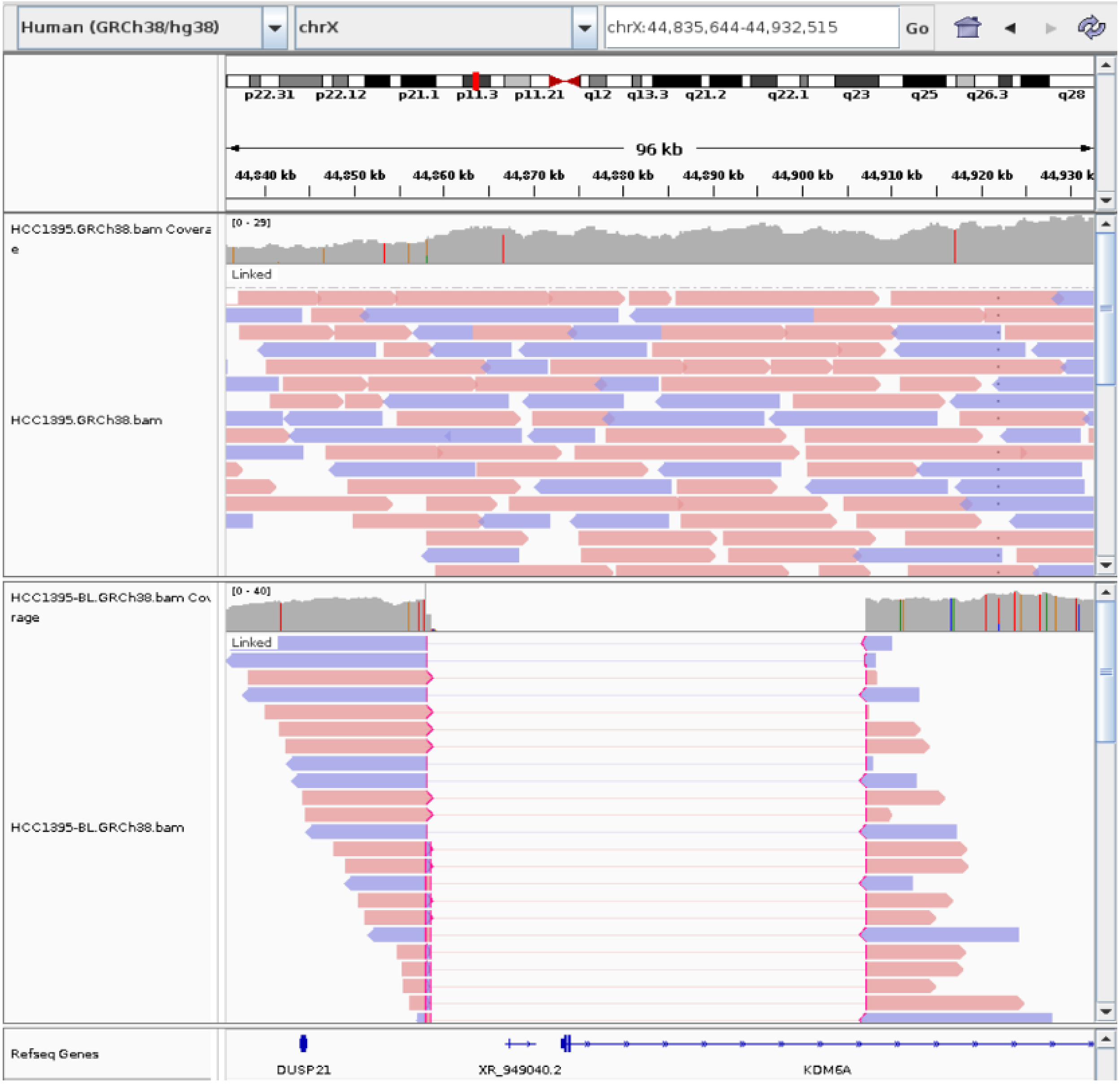

Supplementary Figure 7: A DEL uniquely detected by gSV in HCC1395. The DEL is located within the KDM6A exonic region. KDM6A is commonly mutated in multiple cancer types, including gastric, urothelial, pancreatic, and breast cancer. Previous research [14] has shown that silencing KDM6A promoted cell migration and transformation, demonstrated by the formation of tumor-like acini in three-dimensional culture.

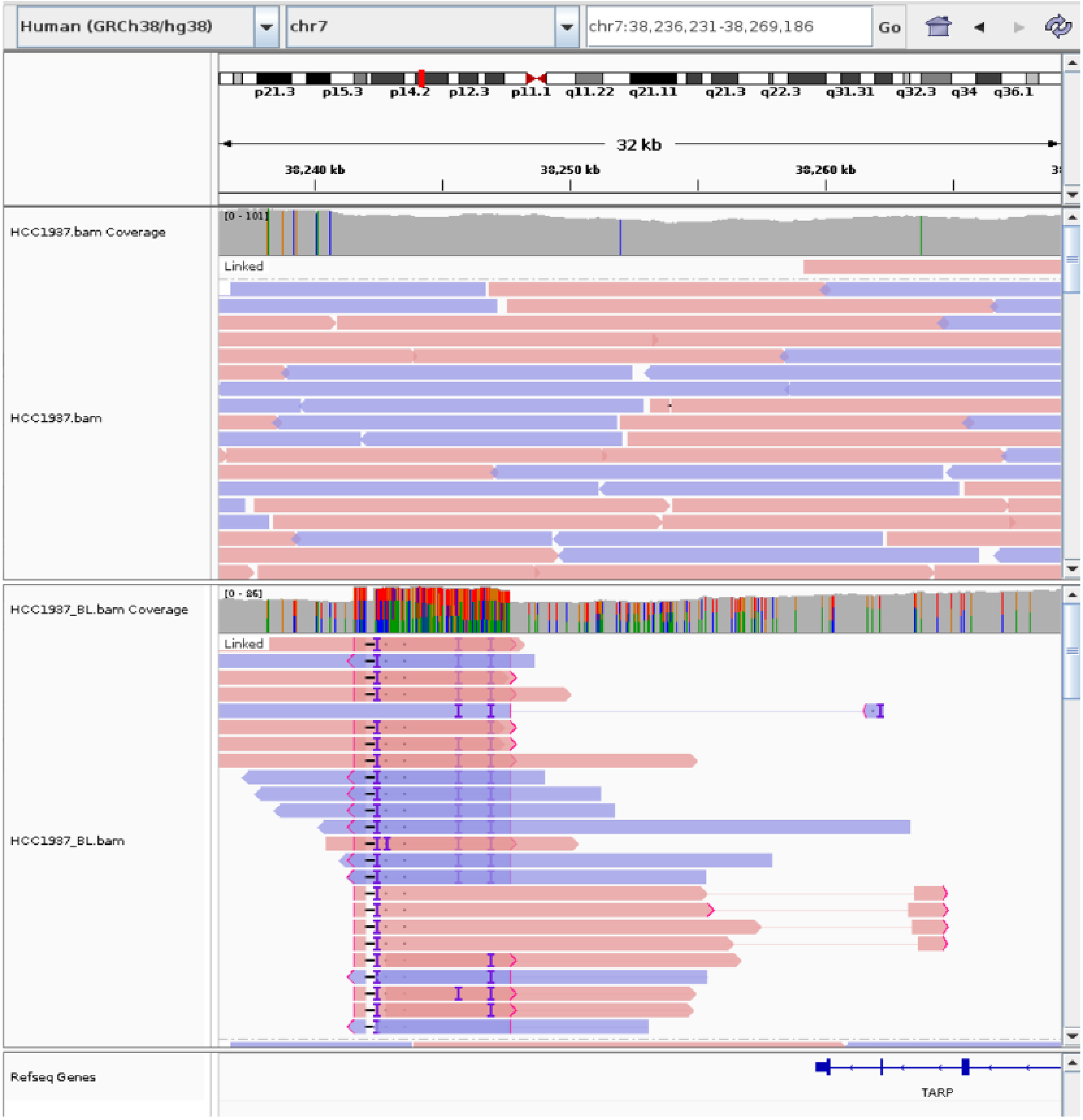

Supplementary Figure 8: A DUP uniquely detected by gSV in HCC1937. The DUP is located within the exonic region of the TCR gamma alternate reading frame protein (TARP, a nuclear protein expressed in prostate and breast cancer cells [15]).

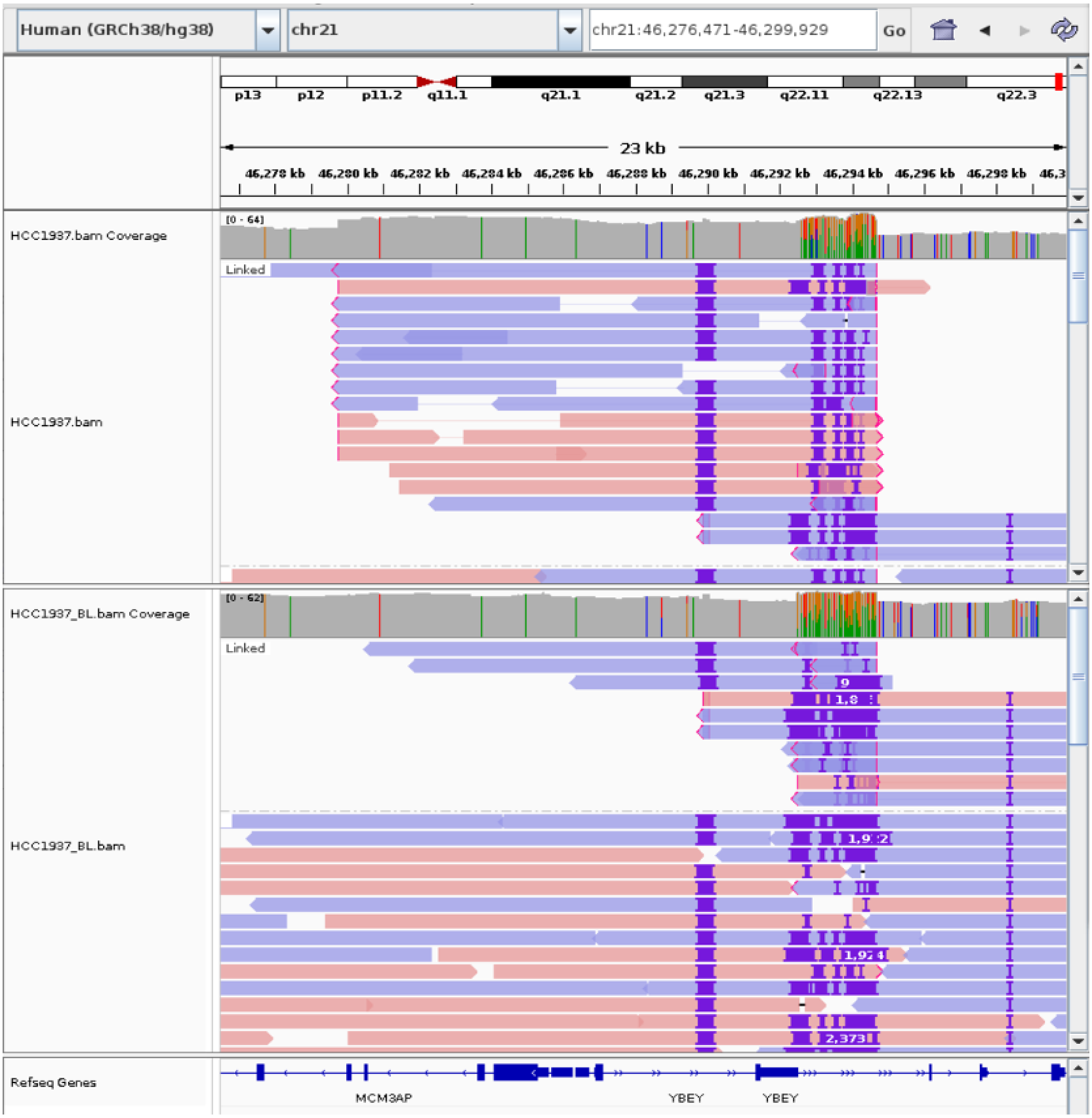

Supplementary Figure 9: A DUP uniquely detected by gSV in HCC1937. The DUP is located within the MCM3AP exonic region. Previous studies have shown that the lncRNA MCM3AP-AS1 may be a novel breast cancer lncRNA with high expression levels in breast cancer patients' tissue [16]. It can promote breast cancer progression via modulating the miR-28-5p/CENPF axis [17].

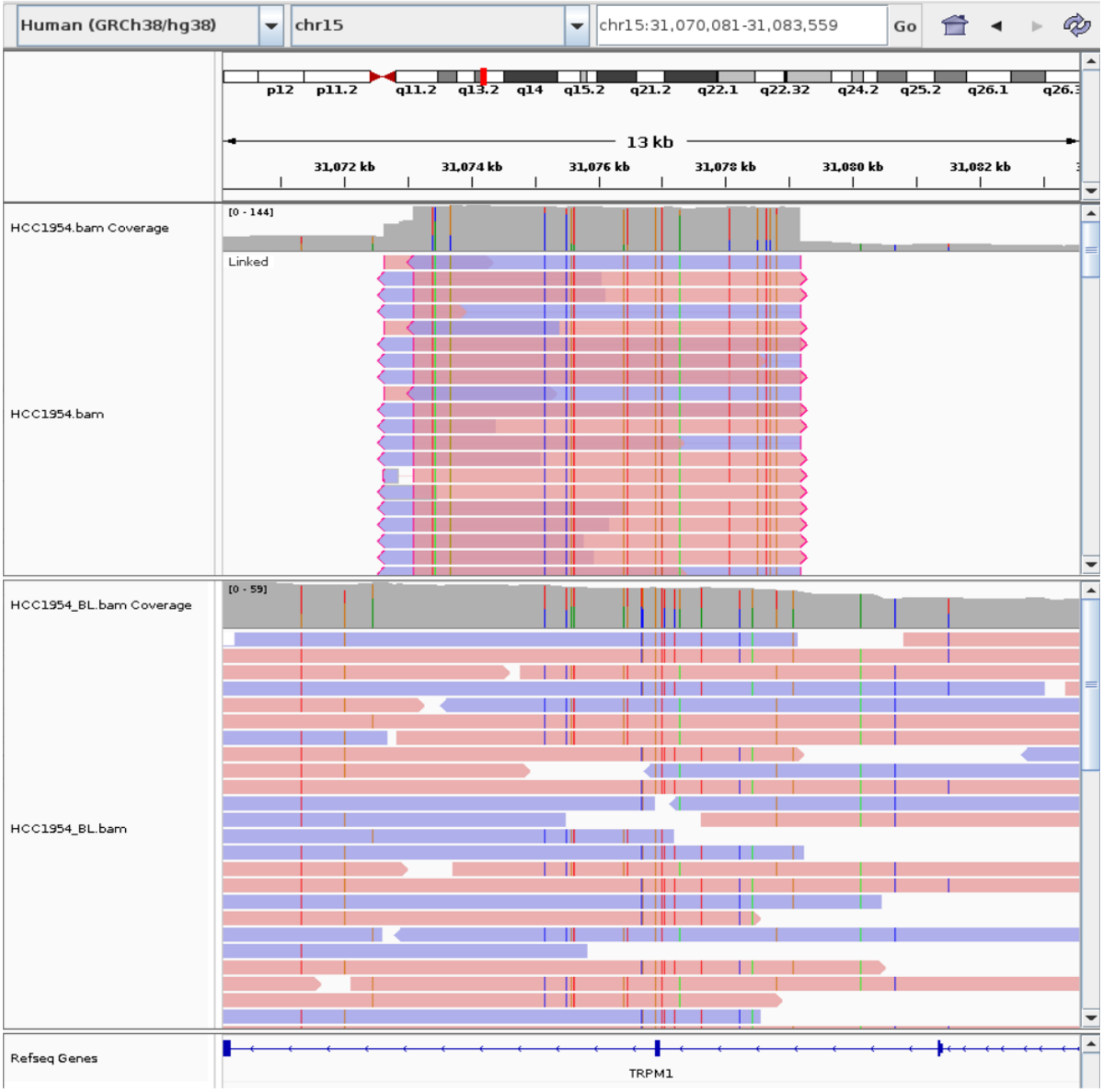

Supplementary Figure 10: A somatic complex SV (DUP+invDUP) uniquely detected by gSV in HCC1954. The complex SV is located within the exonic region of TRPM1. TRPM1 is a specific gene for breast carcinoma [18] and can be used as a great diagnostic tool, especially for Triple-negative Breast Cancer (TNBC).

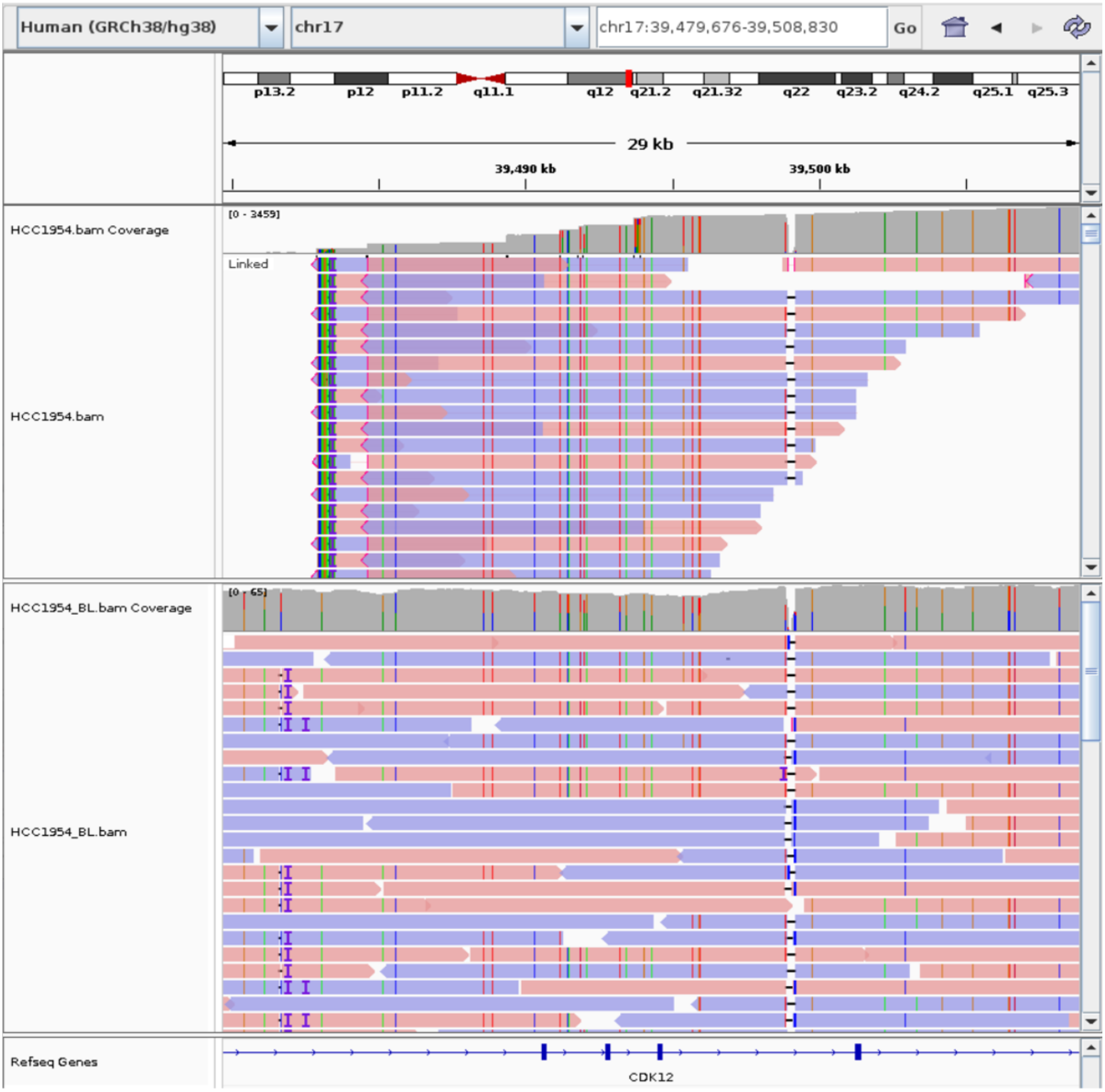

Supplementary Figure 11: A somatic complex SV (DUP+invDUP) uniquely detected by gSV in HCC1954. The complex SV is located within the exonic region of CDK12, which is a primary oncogene. When CDK12 is overexpressed in the normal mammary epithelium, it leads to the formation of spontaneous breast tumors, in addition to actively cooperating in chemical carcinogen- or oncogene-induced breast tumorigenesis [19].

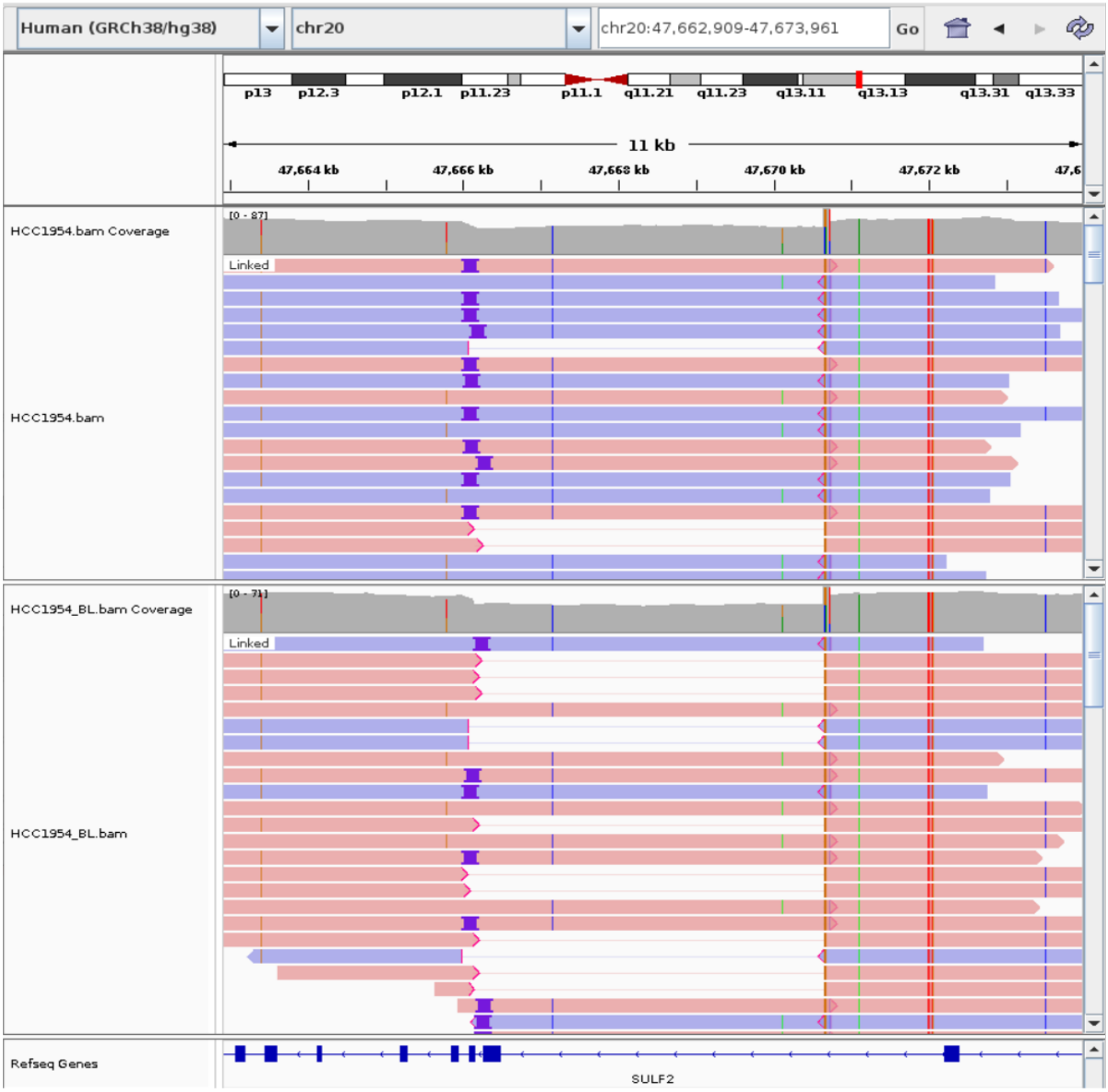

Supplementary Figure 12: A DEL uniquely detected by gSV in HCC1954. The DEL is located within the SULF2 exonic region. Previous research [20] has shown that SULF2 increased breast cancer proliferation, invasion, mobility, and adhesion both in vitro and in vivo.

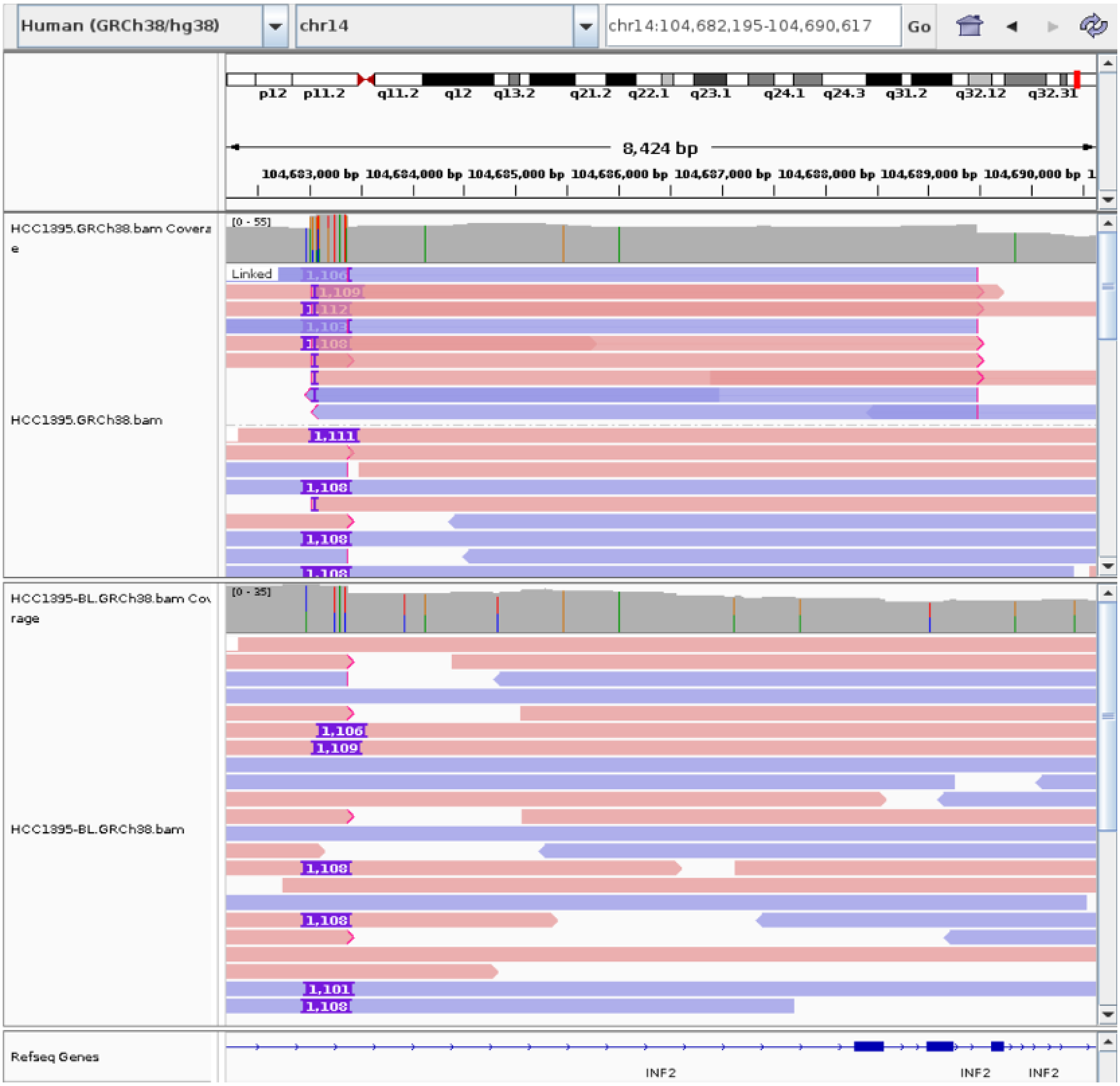

Supplementary Figure 13: A DUP uniquely detected by gSV in HCC1395. The DUP is located within the upstream regulatory region of INF2. Previous research [21] has shown that basal-like breast cancers frequently overexpress formin proteins FHOD1 and INF2.

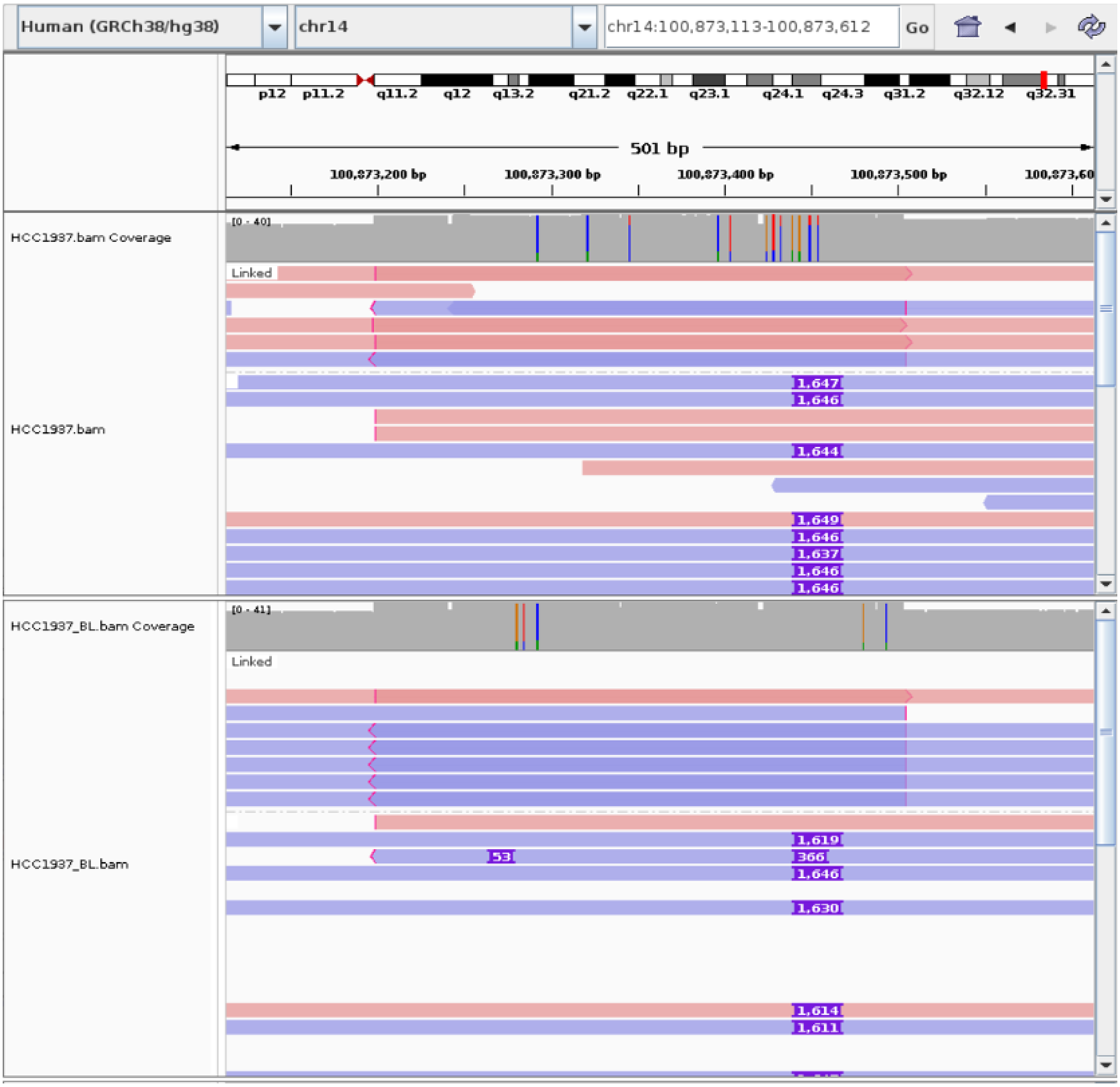

Supplementary Figure 14: A DUP uniquely detected by gSV in HCC1937. The DUP is located within the upstream regulatory region of MIR337. Previous research [22] has shown that this microRNA can suppress breast cancer cell migration and invasion via downregulation of ESRP1.

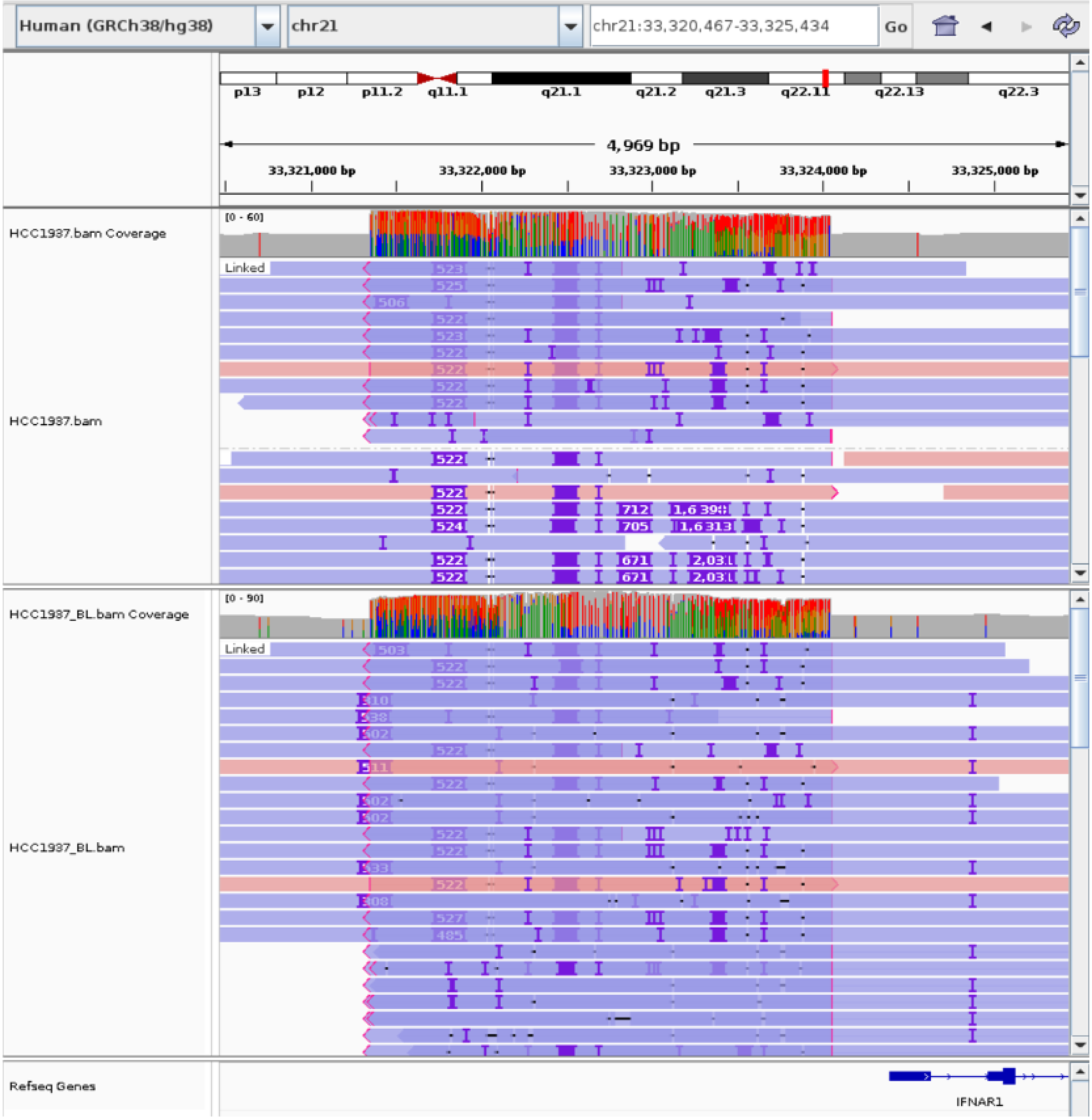

Supplementary Figure 15: A DUP uniquely detected by gSV in HCC1937. The DUP is located within the upstream regulatory region of IFNAR1. Previous research [23] has shown that high IFNAR1 levels in breast cancer cells are associated with poor prognosis.

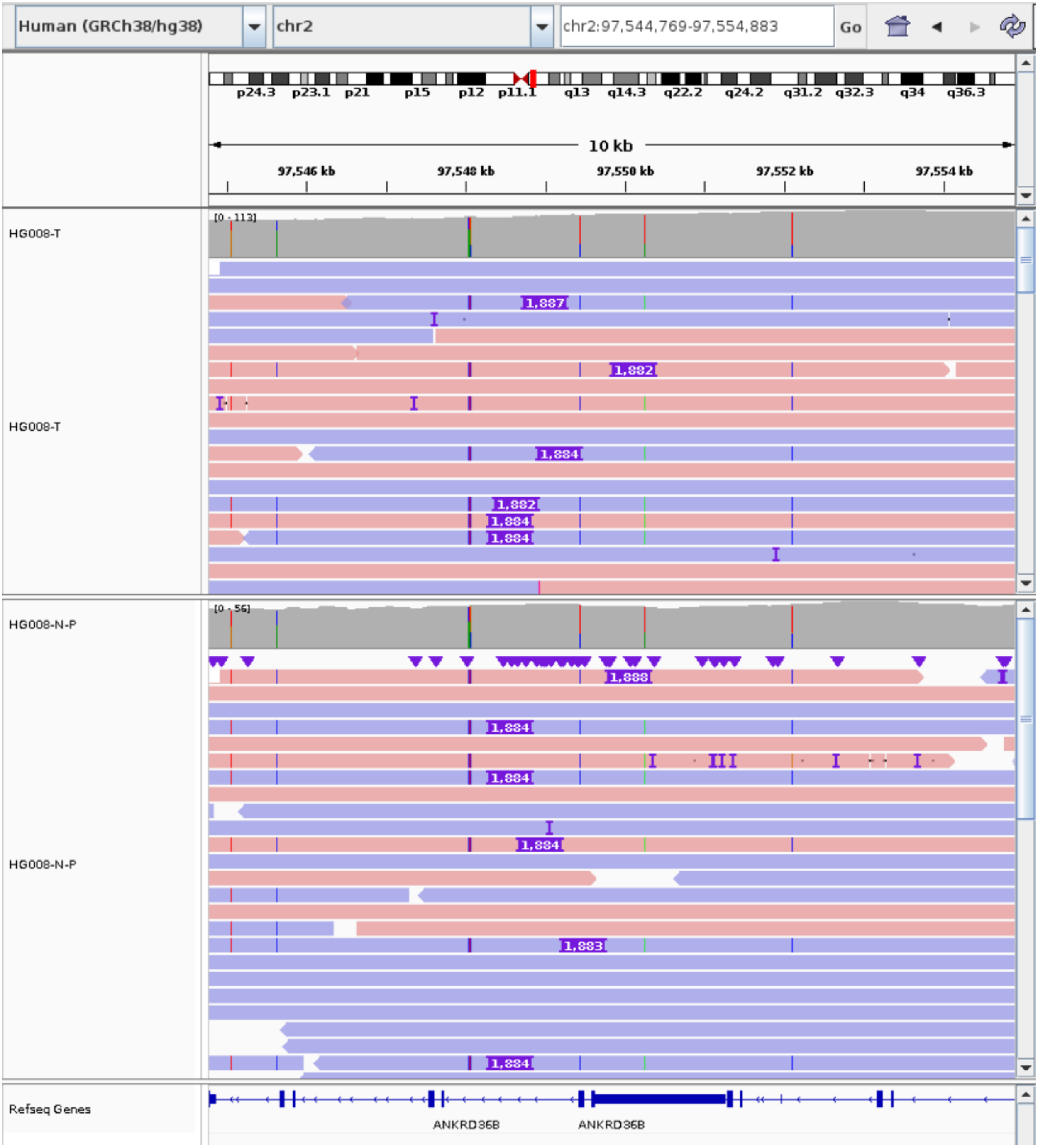

Supplementary Figure 16: A DUP uniquely detected by gSV in HG008. The DUP is located within the exonic region of ANKRD36B. The role of ANKRD36B in cancer is less well-established, but the Human Protein Atlas suggests it's expressed in various cancer tissues.

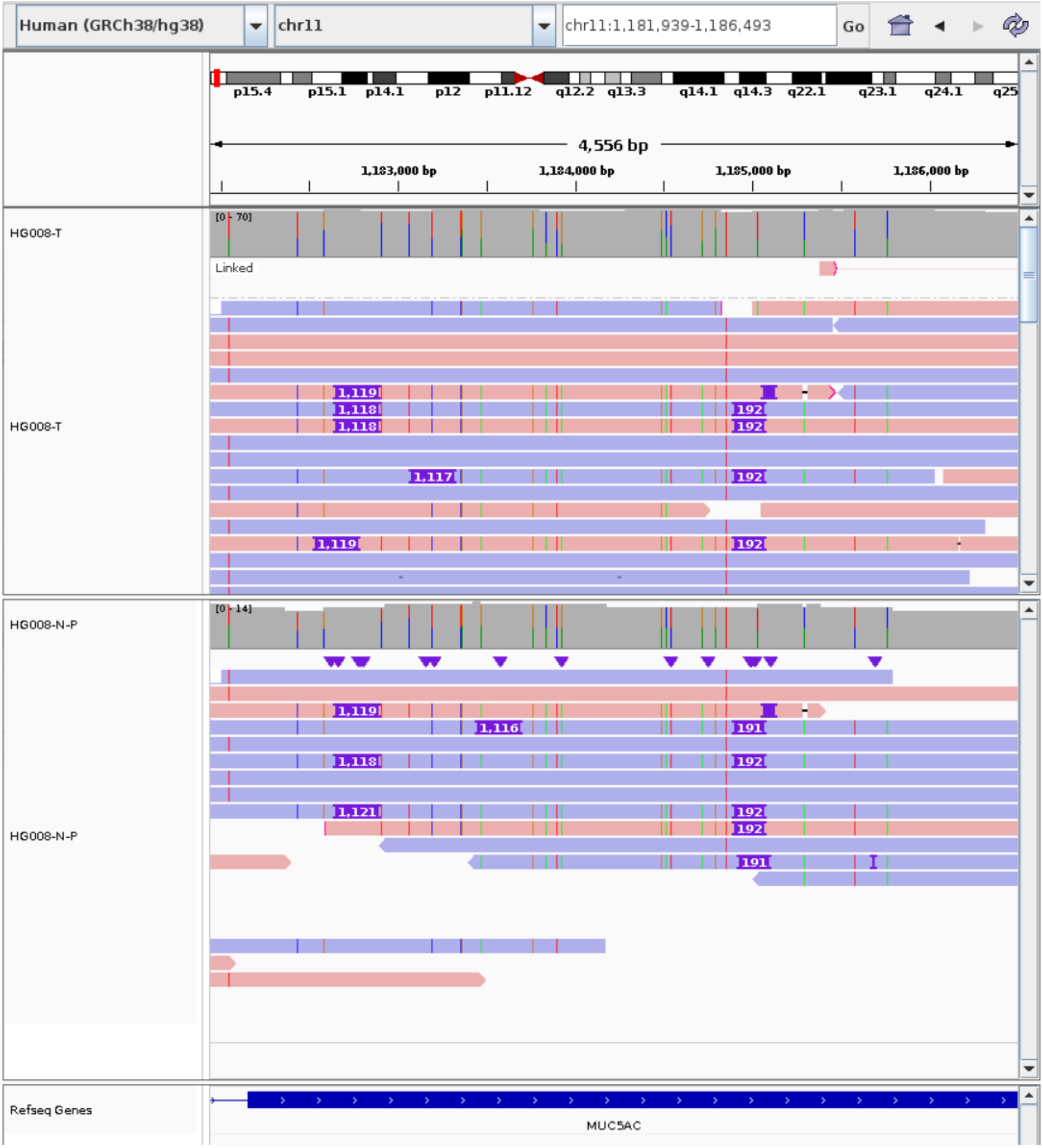

Supplementary Figure 17: A DUP uniquely detected by gSV in HG008. The DUP is located within the exonic region of MUC5AC. Previous research [24] has shown that MUC5AC is a secretory mucin aberrantly expressed in various cancers, e.g. lung cancer, but the relationship with breast cancer is unclear.

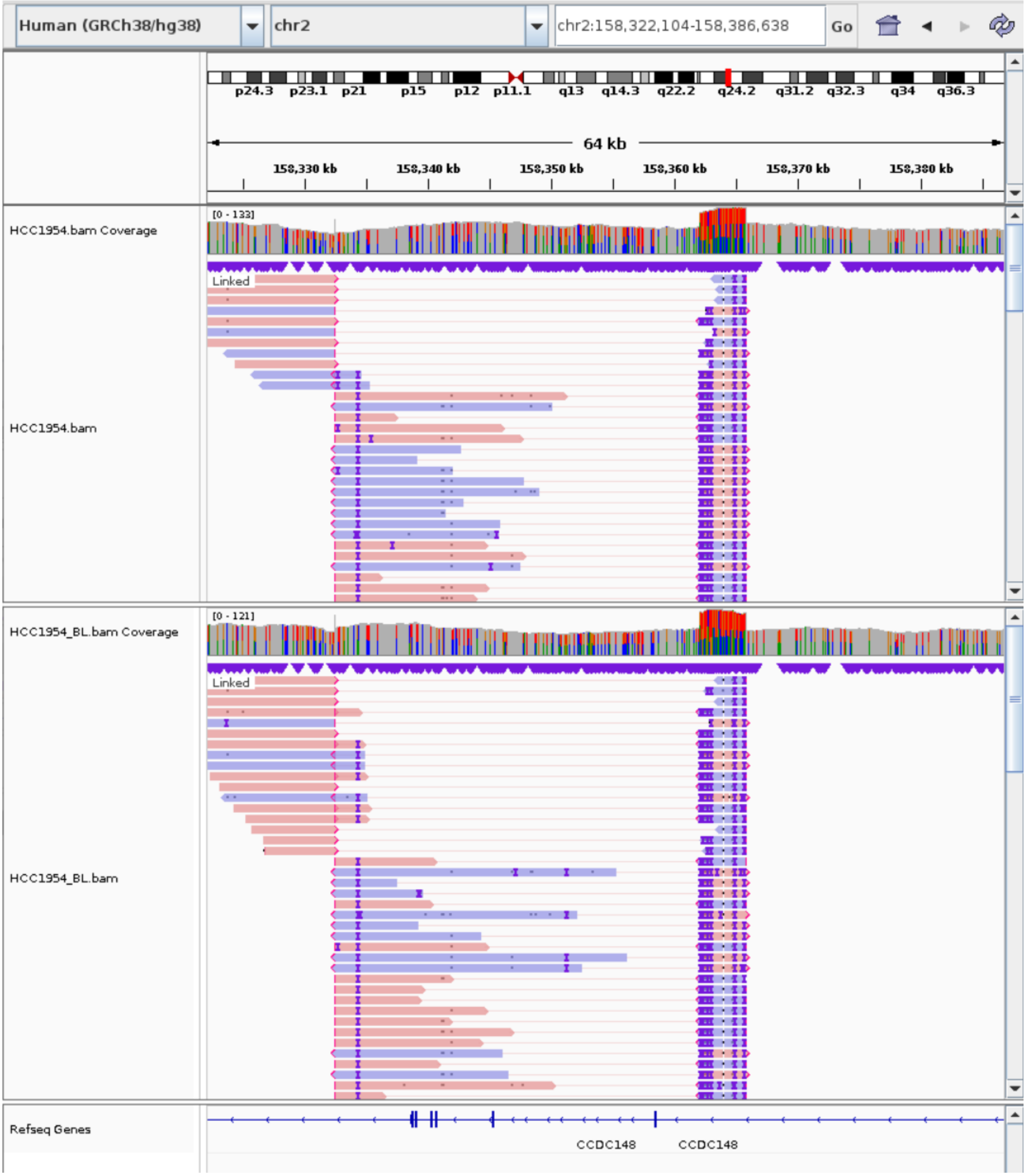

Supplementary Figure 18: An INV uniquely detected by gSV in HCC1954. The INV is located within the exonic region of CCDC148. Previous research [25] has found that CCDC148 gene variants are associated with the risk of aromatase inhibitors-induced musculoskeletal syndrome (AIMSS) in HR+ breast cancer patients treated with aromatase inhibitors. Further research is needed to discover and validate genetic predictors of AIMSS.

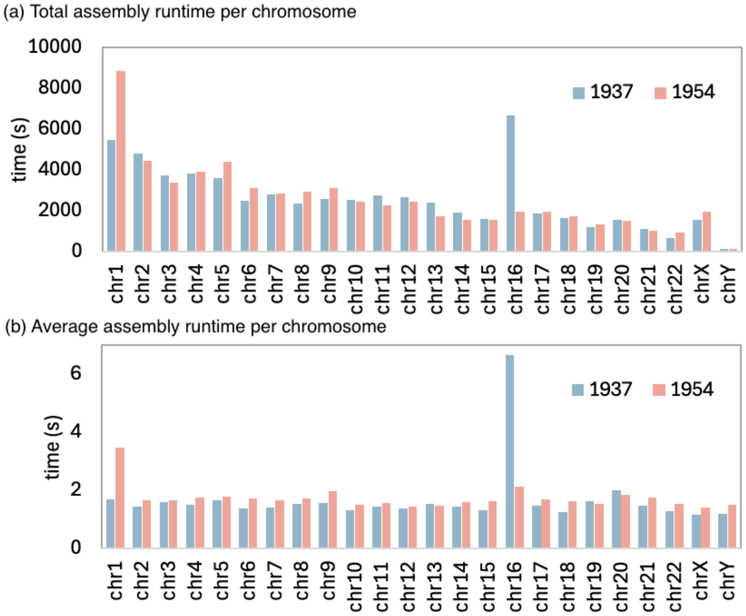

Supplementary Figure 19: Runtime analysis of chromosome-level assembly in HCC1937 and HCC1954. The average assembly time per region is approximately 1.6 seconds. For most chromosomes, runtime scales approximately proportionally with chromosome length, indicating stable computational behavior. However, an exception is observed for chromosome 16 in HCC1937, where the runtime is significantly higher than expected. This increase is driven by a small number of highly complex regions, for which the assembly of a single region can exceed 30 minutes, thereby substantially elevating the total runtime. Notably, this phenomenon appears to be sample-specific, as it is not observed for chromosome 16 in HCC1954.

References

[1] J. C. Mu, M. Mohiyuddin, J. Li, N. Bani Asadi, M. B. Gerstein, A. Abyzov, W. H. Wong, H. Y. Lam, Varsim: a high-fidelity simulation and validation framework for high-throughput genome sequencing with cancer applications, Bioinformatics 31 (9) (2015) 1469–1471.

[2] D. Bolognini, A. Sanders, J. O. Korbel, A. Magi, V. Benes, T. Rausch, Visor: a versatile haplotype-aware structural variant simulator for short-and long-read sequencing, Bioinformatics 36 (4) (2020) 1267–1269.

[3] Y. Ono, K. Asai, M. Hamada, Pbsim2: a simulator for long-read sequencers with a novel generative model of quality scores, Bioinformatics 37 (5) (2021) 589–595.

[4] A. C. English, V. K. Menon, R. A. Gibbs, G. A. Metcalf, F. J. Sedlazeck, Truvari: refined structural variant comparison preserves allelic diversity, Genome Biology 23 (1) (2022) 271.

[5] F. J. Sedlazeck, P. Rescheneder, M. Smolka, H. Fang, M. Nattestad, A. Von Haeseler, M. C. Schatz, Accurate detection of complex structural variations using single-molecule sequencing, Nature Methods 15 (6) (2018) 461–468.

[6] T. Jiang, Y. Liu, Y. Jiang, J. Li, Y. Gao, Z. Cui, Y. Liu, B. Liu, Y. Wang, Long-read-based human genomic structural variation detection with cuteSV, Genome Biology 21 (1) (2020) 1–24.

[7] Y. Chen, A. Y. Wang, C. A. Barkley, Y. Zhang, X. Zhao, M. Gao, M. D. Edmonds, Z. Chong, Deciphering the exact breakpoints of structural variations using long sequencing reads with DeBreak, Nature Communications 14 (1) (2023) 283.

[8] S. Wang, J. Lin, P. Jia, T. Xu, X. Li, Y. Liu, D. Xu, S. Bush, D. Meng, K. Ye, De novo and somatic structural variant discovery with SVision-pro, Nature Biotechnology (2024) https://doi.org/10.1038/s41587–024–02190–7.

[9] M. Kirsche, G. Prabhu, R. Sherman, B. Ni, A. Battle, S. Aganezov, M. C. Schatz, Jasmine and iris: population-scale structural variant comparison and analysis, Nature Methods 20 (3) (2023) 408–417.

[10] D. C. Jeffares, C. Jolly, M. Hoti, D. Speed, L. Shaw, C. Rallis, F. Balloux, C. Dessimoz, J. Bähler, F. J. Sedlazeck, Transient structural variations have strong effects on quantitative traits and reproductive isolation in fission yeast, Nature Communications 8 (1) (2017) 14061.

[11] W.-j. Shen, Y. Zhang, Rpn1 promotes the proliferation and invasion of breast cancer cells by activating the pi3k/akt/mtor signaling pathway, Discover Oncology 15 (1) (2024) 25.

[12] S. Matsukita, M. Nomoto, S. Kitajima, S. Tanaka, M. Goto, T. Irimura, Y. Kim, E. Sato, S. Yonezawa, Expression of mucins (muc1, muc2, muc5ac and muc6) in mucinous carcinoma of the breast: comparison with invasive ductal carcinoma, Histopathology 42 (1) (2003) 26–36.

[13] C. De Bolòs, M. Gumà, C. Barranco, M. Garrido, Y. S. Kim, F. X. Real, Muc6 expression in breast tissues and cultured cells: abnormal expression in tumors and regulation by steroid hormones, International Journal of Cancer 77 (2) (1998) 193–199.

[14] J.-F. Xiao, L.-F. Kua, L.-W. Ding, Q.-Y. Sun, K. N. Myint, X.-R. Chia, N. Venkatachalam, X. Loh, J. E. Duex, V. Neang, et al., Kdm6a depletion in breast epithelial cells leads to reduced sensitivity to anticancer agents and increased tgfβ activity, Molecular Cancer Research 20 (4) (2022) 637–649.

[15] C. D. Wolfgang, M. Essand, J. J. Vincent, B. Lee, I. Pastan, Tarp: A nuclear protein expressed in prostate and breast cancer cells derived from an alternate reading frame of the t cell receptor γ chain locus, Proceedings of the National Academy of Sciences 97 (17) (2000) 9437–9442.

[16] T.-P. Tang, C.-X. Qin, H. Yu, Mcm3ap-as1 regulates proliferation, apoptosis, migration, and invasion of breast cancer cells via binding with zfp36, Translational Cancer Research 10 (10) (2021) 4478.

[17] Q. Chen, H. Xu, J. Zhu, K. Feng, C. Hu, Lncrna mcm3ap-as1 promotes breast cancer progression via modulating mir-28-5p/cenpf axis, Biomedicine & Pharmacotherapy 128 (2020) 110289.

[18] D. Ai, J. Yao, F. Yang, L. Huo, H. Chen, W. Lu, L. M. S. Soto, M. Jiang, M. G. Raso, S. Wang, et al., Trps1: a highly sensitive and specific marker for breast carcinoma, especially for triple-negative breast cancer, Modern Pathology 34 (4) (2021) 710–719.

[19] M. Filippone, D. Gaglio, R. Bonfanti, F. Tucci, E. Ceccacci, R. Pennisi, M. Bonanomi, G. Jodice, M. Tillhon, F. Montani, et al., Cdk12 promotes tumorigenesis but induces vulnerability to therapies inhibiting folate one-carbon metabolism in breast cancer, Nature Communications 13 (1) (2022) 2642.

[20] C. Zhu, L. He, X. Zhou, X. Nie, Y. Gu, Sulfatase 2 promotes breast cancer progression through regulating some tumor-related factors, Oncology Reports 35 (3) (2016) 1318–1328.

[21] V. D. Heuser, N. Mansuri, J. Mogg, S. Kurki, H. Repo, P. Kronqvist, O. Carpén, M. Gardberg, Formin proteins fhod1 and inf2 in triple-negative breast cancer: association with basal markers and functional activities, Breast Cancer: Basic and Clinical Research 12 (2018) 1178223418792247.

[22] Y. Pan, Y. Zhao, L. Lihui, Y. Xie, Q. Zou, Mir-337-3p suppresses migration and invasion of breast cancer cells by downregulating esrp1, Acta Histochemica 123 (7) (2021) 151777.

[23] O. Odnokoz, P. Yu, A. R. Peck, Y. Sun, A. J. Kovatich, J. A. Hooke, H. Hu, E. P. Mitchell, H. Rui, S. Y. Fuchs, Malignant cell-specific pro-tumorigenic role of type i interferon receptor in breast cancers, Cancer biology & therapy 21 (7) (2020) 629–636.

[24] I. Lakshmanan, S. Rachagani, R. Hauke, S. Krishn, S. Paknikar, P. Seshacharyulu, S. Karmakar, R. Nimmakayala, G. Kaushik, S. Johansson, et al., Muc5ac interactions with integrin β4 enhances the migration of lung cancer cells through fak signaling, Oncogene 35 (31) (2016) 4112–4121.

[25] Y. Liang, C. L. Gersch, J. Lehman, N. L. Henry, K. L. Smith, J. M. Rae, V. Stearns, D. L. Hertz, Attempted replication of pharmacogenetic association of variants in ppp1r14c and ccdc148 with aromatase inhibitor-induced musculoskeletal symptoms, Pharmacogenetics and Genomics 34 (4) (2024) 126–129.
